# CRP-Like Transcriptional Regulator MrpC Curbs c-di-GMP and 3′,3′-cGAMP Nucleotide Levels during Development in Myxococcus xanthus

**DOI:** 10.1128/mbio.00044-22

**Published:** 2022-02-15

**Authors:** Sofya Kuzmich, Patrick Blumenkamp, Doreen Meier, Dobromir Szadkowski, Alexander Goesmann, Anke Becker, Lotte Søgaard-Andersen

**Affiliations:** a Department of Ecophysiology, Max Planck Institute for Terrestrial Microbiology, Marburg, Germany; b Systems Biology and Bioinformatics, Justus Liebig University Giessen, Gissen, Germany; c Center for Synthetic Microbiology (SYNMIKRO), Philipps Universität Marburg, Marburg, Germany; Institut Pasteur

**Keywords:** c-di-GMP, cGAMP, CRP, Cappable-seq, sporulation, fruiting body formation, development, diguanylate cyclase, phosphodiesterase, PilZ, second messenger, 3', 3'-cGAMP, CRP-like proteins, cyclic nucleotides

## Abstract

Myxococcus xanthus has a nutrient-regulated biphasic life cycle forming predatory swarms in the presence of nutrients and spore-filled fruiting bodies in the absence of nutrients. The second messenger 3′-5′, 3′-5 cyclic di-GMP (c-di-GMP) is essential during both stages of the life cycle; however, different enzymes involved in c-di-GMP synthesis and degradation as well as several c-di-GMP receptors are important during distinct life cycle stages. To address this stage specificity, we determined transcript levels using transcriptome sequencing (RNA-seq) and transcription start sites using Cappable sequencing (Cappable-seq) during growth and development genome wide. All 70 genes encoding c-di-GMP-associated proteins were expressed, with 28 upregulated and 10 downregulated during development. Specifically, the three genes encoding enzymatically active proteins with a stage-specific function were expressed stage specifically. By combining operon mapping with published chromatin immunoprecipitation sequencing (ChIP-seq) data for MrpC (M. Robinson, B. Son, D. Kroos, L. Kroos, BMC Genomics 15:1123, 2014, http://dx.doi.org/10.1186/1471-2164-15-1123), the cAMP receptor protein (CRP)-like master regulator of development, we identified nine developmentally regulated genes as regulated by MrpC. In particular, MrpC directly represses the expression of *dmxB*, which encodes the diguanylate cyclase DmxB that is essential for development and responsible for the c-di-GMP increase during development. Moreover, MrpC directly activates the transcription of *pmxA*, which encodes a bifunctional phosphodiesterase that degrades c-di-GMP and 3′,3′-cGAMP *in vitro* and is essential for development. Thereby, MrpC regulates and curbs the cellular pools of c-di-GMP and 3′,3′-cGAMP during development. We conclude that temporal regulation of the synthesis of proteins involved in c-di-GMP metabolism contributes to c-di-GMP signaling specificity. MrpC is important for this regulation, thereby being a key regulator of developmental cyclic di-nucleotide metabolism in M. xanthus.

## INTRODUCTION

In bacteria, signaling by nucleotide-based second messengers has important functions in adaptive responses to environmental changes (1–6). Notably, 3′-5′, 3′-5 cyclic di-GMP (c-di-GMP) is a versatile second messenger that regulates numerous processes, including exopolysaccharide synthesis, biofilm formation, cell cycle progression, virulence, motility, and multicellular development (1, 2). c-di-GMP is synthesized by diguanylate cyclases (DGCs), which contain the conserved GGDEF domain, and degraded by phosphodiesterases (PDEs), which contain an EAL or HD-GYP domain (1, 2). The effects of changing c-di-GMP levels are implemented by c-di-GMP binding receptors, which regulate downstream responses at the transcriptional, translational, or posttranslational level (1, 2). Reflecting c-di-GMP versatility, c-di-GMP receptors comprise a variety of proteins with little sequence homology, including enzymatically inactive DGC and EAL domain proteins (7–11), PilZ domain proteins (12–16), MshEN domain proteins (17, 18), and proteins of different transcription factor families (19–26). Among these receptors, enzymatically inactive DGC and EAL domain proteins as well as PilZ and MshEN domains can be predicted bioinformatically (17, 27).

Often, individual bacterial genomes encode multiple DGCs, PDEs, and c-di-GMP receptors (1). Yet, the inactivation of individual genes for DGCs, PDEs, and c-di-GMP receptors can result in distinct phenotypes, underscoring that specific signaling modules exist. Thus, a central question is how this signaling specificity is accomplished. Three mutually nonexclusive models have been proposed to explain this specificity (1, 28, 29). First, individual signaling modules can be separated temporally based on the differential regulation of their synthesis and/or degradation; second, individual signaling modules can be separated spatially by protein complex formation or by localizing to distinct subcellular locations; and, third, effectors of different signaling modules may have different binding affinities for c-di-GMP.

Myxococcus xanthus is a model organism for studying social behaviors and cell differentiation in bacteria (30). M. xanthus has a nutrient-regulated biphasic life cycle. In the presence of nutrients, cells form predatory swarms that spread coordinately using type IV pilus (T4P)-dependent motility and gliding motility (31, 32). Upon nutrient depletion, M. xanthus initiates a developmental program that culminates in the formation of multicellular, spore-filled fruiting bodies, while cells that remain outside fruiting bodies differentiate to either so-called peripheral rods or undergo cell lysis (33–35). Nucleotide-based second messengers have important roles during both stages of the life cycle. During growth, c-di-GMP is important for type IV pili-dependent motility and regulation of motility (36, 37). During development, the starvation-induced activation of the stringent response with synthesis of the second messenger (p)ppGpp is required and sufficient to initiate development (38, 39). Moreover, the cellular c-di-GMP level increases dramatically during development, and this increase is essential for the completion of development (40). Development also depends on global transcriptional changes (41), regulation of motility (31, 32), and cell-cell signaling (30, 42).

Several transcription factors that are essential for fruiting body formation and sporulation have been identified (41). Among these factors, MrpC is a member of the cAMP receptor protein (CRP) family of transcription factors (43) and has been referred to as a master regulator of development (41). Currently, no ligand for MrpC has been reported, and MrpC on its own binds target promoters *in vitro* (44–51). MrpC alone is a negative autoregulator (44) and directly activates the transcription of *fruA* (45), which encodes a transcriptional regulator that is also essential for development (52, 53). MrpC and FruA jointly regulate the expression of multiple genes during development (46–51).

Systematic inactivation of all 36 genes for GGDEF domain proteins, EAL domain proteins, and HD-GYP domain proteins identified only three enzymatically active proteins that are important during growth and development under standard laboratory conditions. Interestingly, each of the three proteins are important during a distinct stage of the life cycle. The DGC DmxA is important for T4P-dependent motility in the presence of nutrients but not for development (37, 40). In contrast, the DGC DmxB and the HD-GYP-type PDE PmxA are important exclusively for development (37, 40). DmxB is the DGC responsible for the dramatic increase in the c-di-GMP level during development (40). PmxA degrades c-di-GMP as well as the di-nucleotide 3′-5′, 3′-5′-cyclic GMP-AMP (cGAMP) *in vitro* and with the highest activity toward cGAMP (40, 54). The lack of PmxA does not lead to significant changes in the c-di-GMP level during development (40), while it remains unknown how a lack of PmxA may affect cGAMP accumulation *in vivo*. The GacA and GacB proteins were analyzed *in vitro* and shown to belong to the Hypr subfamily of GGDEF domain proteins that synthesize cGAMP rather than c-di-GMP (55).

Several c-di-GMP receptors have been verified experimentally in M. xanthus. The histidine protein kinase SgmT contains an enzymatically inactive GGDEF domain that binds c-di-GMP and works together with the DNA binding response regulator DigR to regulate extracellular matrix composition during growth and development (8, 37). The enhancer binding protein Nla24 also binds c-di-GMP and is important for motility during growth as well as development (40, 56, 57). Systematic inactivation of all 24 genes encoding PilZ domain proteins identified PixA and PixB as c-di-GMP receptors that regulate motility (36). While PixA is important only during growth, PixB is crucial during growth and development (36). Finally, the ribbon-helix-helix proteins CdbA and CdbB bind c-di-GMP (58). CdbA is an essential nucleoid-associated protein important for chromosome organization and segregation (58).

With the exception of DmxB, its synthesis of which is strongly upregulated during development (40), it is not understood how c-di-GMP metabolizing enzymes and some verified receptors are functionally restricted to either growth or development. To increase our understanding of c-di-GMP signaling and specificity in M. xanthus, we used transcriptome sequencing (RNA-seq) to determine during which stage(s) of the life cycle the 70 genes encoding c-di-GMP metabolizing enzymes, potential c-di-GMP receptors, and known c-di-GMP receptors (from here on “c-di-GMP-associated proteins”) are expressed. We found that all of these genes are expressed, with 28 being upregulated and 10 downregulated during development. In particular, transcription of the three genes encoding enzymatically active proteins with a stage-specific function were regulated in a stage-specific manner, supporting that temporal regulation of the synthesis of proteins involved in c-di-GMP metabolism contributes to signaling specificity. To inform the RNA-seq analysis, we performed Cappable sequencing (Cappable-seq) to identify transcription start sites (TSSs) at a genome-wide scale. These data together with a previously published chromatin immunoprecipitation sequencing (ChIP-seq) analysis to map MrpC binding sites during development (50) revealed nine of the developmentally regulated genes as candidates for being directly regulated by MrpC. In particular, we found that MrpC directly represses *dmxB* and activates *pmxA* expression. Consistently, a Δ*mrpC* mutant has an increased accumulation of c-di-GMP and cGAMP.

## RESULTS

### RNA-seq profiling reveals pervasive developmental regulation of genes encoding c-di-GMP-associated proteins.

To elucidate whether transcriptional regulation of genes for c-di-GMP-associated proteins contributes to their stage-specific function, we performed RNA-seq analyses using the wild-type (WT) strain DK1622. To this end, we collected total RNA from nonstarved cells (from here on referred to as 0 h of development) and from cells developed for 6, 12, 18, and 24 h under submerged culture conditions. These time points span the entire process of the aggregation of cells to the formation of fruiting bodies and the early stages of sporulation. RNA was isolated from two biological replicates. RNA sample preparation, depletion of rRNA, sequencing, and data analysis are described in the Materials and Methods. Benchmarking of the RNA-seq data using reverse transcription-quantitative PCR (RT-qPCR) analyses of the *mrpC* and *fruA* genes that are both transcriptionally upregulated during development (43, 44, 52, 53) demonstrated that the two genes had the same expression patterns in the two approaches (see Fig. S1 in the supplemental material).

**FIG 1:**
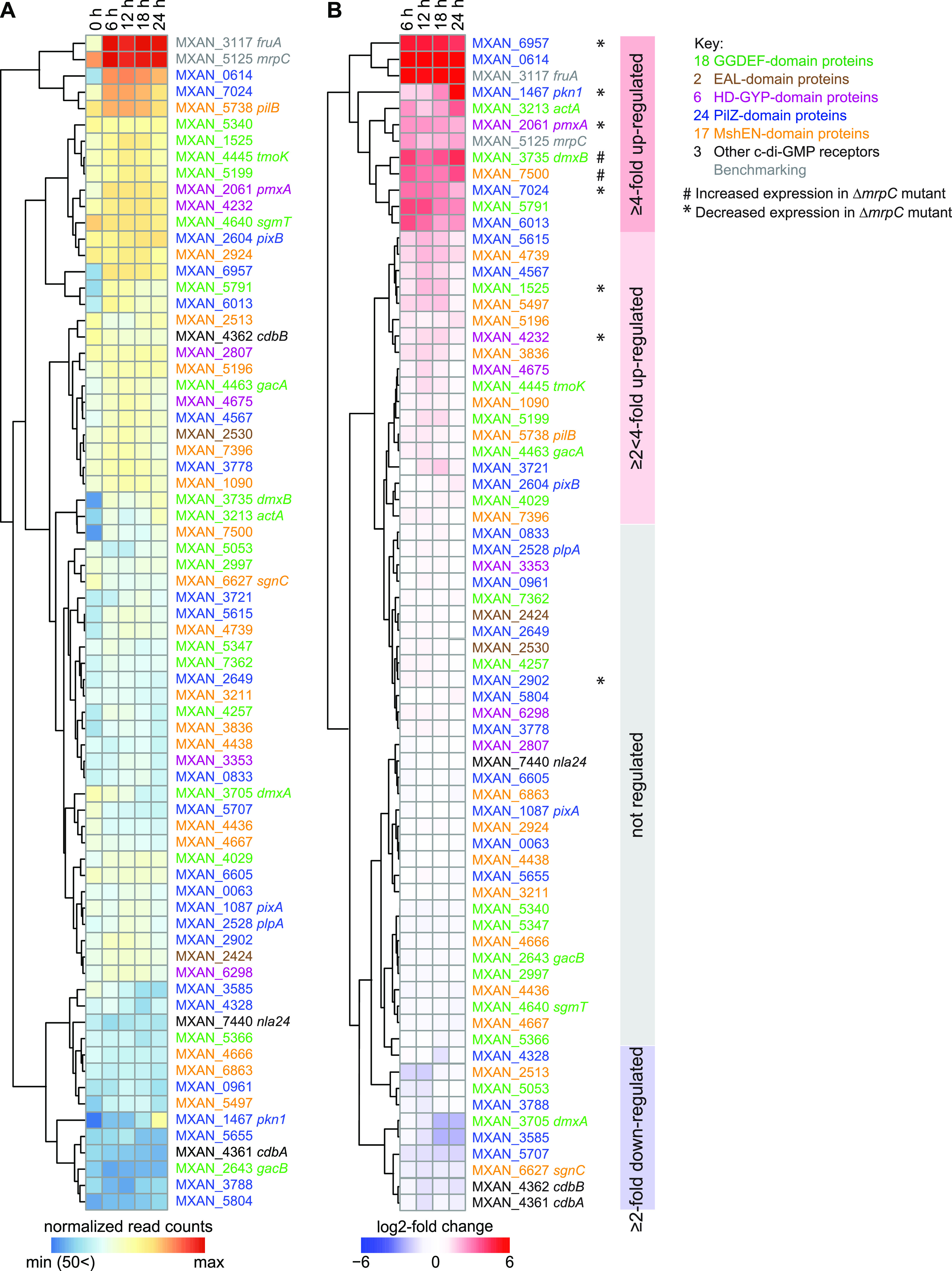
Expression of genes for “c-di-GMP-associated proteins.” (A) Expression of the genes encoding c-di-GMP-associated proteins. The heatmap shows normalized read counts at the indicated time points. Genes are color-coded according to the key on the right. MXAN_2807 is indicated as a protein with an HD-GYP domain; this protein also contains a MshEN domain. (B) Relative transcript levels during development for genes encoding c-di-GMP-associated proteins. The heatmap shows the log_2_-fold change at 6, 12, 18, or 24 h of development relative to 0 h calculated from the normalized read counts (Table S1B). Genes marked * or # were expressed at lower and higher levels, respectively, in the Δ*mrpC* mutant compared with those in the WT, as determined using RT-qPCR (see also Fig. 2 and Fig. S3). Colored boxes on the right indicate the four clusters with distinct expression profiles.

10.1128/mbio.00044-22.1 FIG S1 Benchmarking of RNA-seq expression data using RT-qPCR. Total RNA was isolated from WT cells developed in MC7 submerged cultures at the indicated time points. Transcript levels are shown as log_2_-fold change at 6, 12, 18, or 24 h of development relative to 0 h. For RNA-seq data, values were calculated as the ratio of normalized read counts relative to 0 h (Table S1B). RT-qPCR data are shown as mean ± SD from two biological replicates, each with two technical replicates, relative to 0 h. The same RNA samples were used in RNA-seq and RT-qPCR analyses. The color code is as in Fig. 1. Download FIG S1, EPS file, 1.5 MB. Copyright © 2022 Kuzmich et al. 2022 Kuzmich et al. https://creativecommons.org/licenses/by/4.0/ This content is distributed under the terms of the Creative Commons Attribution 4.0 International license.

Subsequently, we focused on the 70 genes encoding c-di-GMP-associated proteins. These genes encode 18 GGDEF domain proteins, 2 EAL domain proteins, 6 HD-GYP domain proteins, 24 PilZ domain proteins, and 17 MshEN domain proteins, as well as CdbA, CdbB, and Nla24. All proteins with one of these domains were included because nonenzymatic proteins or proteins that do not bind c-di-GMP can still be involved in the regulation of c-di-GMP-dependent processes (11, 59). All 70 genes were expressed with normalized read counts of more than 50 at all 5 time points (Fig. 1A; see Table S1A in the supplemental material). A comparison of normalized read counts during development to that during growth (0 h) revealed four clusters with distinct expression profiles. One cluster of 10 genes, including *dmxB*, *pmxA*, and *pkn1* as well as the benchmarking *mrpC* and *fruA* genes, were induced more than 4-fold (log_2_ fold change [FC], ≥2; adjusted *P* ≤ 0.05) at one or more time points during development (Fig. 1B; Table S1B). Pkn1 is a Ser/Thr protein kinase with a C-terminal PilZ domain and is specifically important for development (36, 60); it is not known whether the PilZ domain binds c-di-GMP. These observations are in agreement with previous findings that *dmxB* and *pkn1* transcription is upregulated during development (40, 60). A second cluster of 18 genes, including *tmoK*, *pixB*, *gacA*, and *pilB*, were induced more than 2-fold (log_2_FC, ≥1; adjusted *P* ≤ 0.05) at one or more time point(s) during development. TmoK is a histidine protein kinase with a C-terminal GGDEF domain and is important for T4P-dependent motility during growth as well as for development; the GGDEF domain does not have DGC activity and does not bind c-di-GMP (37, 40). PilB is the ATPase for T4P extension and contains an N-terminal MshEN domain (17, 61), but it is not known whether it binds c-di-GMP. In the third cluster, 10 genes, including *dmxA*, *cdbA*, and *cdbB*, were downregulated more than 2-fold (log_2_FC, ≥−1; adjusted *P* ≤ 0.05) at one or more time point(s) during development (Fig. 1B). Expression of the remaining 32 genes, including *sgmT*, *pixA*, *plpA*, *gacB*, and *nla24*, were not significantly regulated during development (Fig. 1B; Table S1B). PlpA is a PilZ domain protein that regulates motility during growth but is not important for development and was reported not to bind c-di-GMP *in vitro* (36, 62). Control experiments using RT-qPCR on the same RNA as for RNA-seq for selected genes (*dmxA*, *dmxB*, and *pkn1*) reproduced the RNA-seq data (Fig. S1).

10.1128/mbio.00044-22.7 TABLE S1 RNA-seq analysis of M. xanthus genes encoding c-di-GMP-associated proteins in WT.
Table S1A. Normalized read counts at 0, 6, 12, 18, and 24 h of development for both replicates. Table S1B. log_2_-fold changes at 6, 12, 18, or 24 h of development over 0 h calculated from the normalized read counts. Blue color corresponds to log_2_ FC of <−1, orange to log_2_ FC of >1, and green to adjusted a *P* value of <0.05. Old MXAN and new MXAN refer to the original annotation and the RefSeq NC_008095 annotation of the M. xanthus genome. Download Table S1, XLSX file, 0.04 MB. Copyright © 2022 Kuzmich et al. 2022 Kuzmich et al. https://creativecommons.org/licenses/by/4.0/ This content is distributed under the terms of the Creative Commons Attribution 4.0 International license.

10.1128/mbio.00044-22.3 FIG S3 Expression of genes encoding c-di-GMP-associated proteins potentially regulated by MrpC. Total RNA was isolated from cells developed in MC7 submerged cultures at the indicated time points of WT (black) and the Δ*mrpC* mutant (red). Transcript levels were determined using RT-qPCR and are shown as log_2_-fold change at 6, 12, 18, or 24 h of development relative to 0 h as mean ± SD from two biological replicates, each with two technical replicates, relative to WT at 0 h. Download FIG S3, EPS file, 1.9 MB. Copyright © 2022 Kuzmich et al. 2022 Kuzmich et al. https://creativecommons.org/licenses/by/4.0/ This content is distributed under the terms of the Creative Commons Attribution 4.0 International license.

We conclude that the expression of the genes for the enzymatically active proteins (DmxA, DmxB, and PmxA) with a stage-specific function correlates with that stage of the life cycle. Similarly, the gene for the developmentally important Pkn1 protein is upregulated during development, while the gene for the growth-related PixA was expressed constitutively. Similarly, the genes for the three verified c-di-GMP receptors (SgmT, PixB, and Nla24) that function during both stages of the life cycle were expressed constitutively, while the genes for the essential proteins CdbA and CdbB were downregulated during development. Altogether, these observations support that the transcriptional regulation of genes encoding proteins that act in a stage-specific manner may contribute to temporally restricting their activity.

### Genome-wide mapping of transcription start sites using Cappable-seq.

To further understand the transcriptional regulation of genes for c-di-GMP-associated proteins, we performed genome-wide mapping of transcription start sites (TSSs) with a single-nucleotide resolution using Cappable-seq (63). For this mapping, total RNA was isolated in two biological replicates from growing M. xanthus cells (0 h) and from cells developed for 6, 12, 18, and 24 h under the same conditions as those for the RNA-seq analysis. RNA samples were enriched for primary transcripts with a triphosphate at the 5′ end, and cDNA libraries were generated and sequenced (Materials and Methods). The number of reads starting at a certain position was normalized to the total number of reads to obtain a relative read score (RRS) (Materials and Methods). As in reference 63, TSSs with an RRS of <1.5 (equivalent to ∼10 reads or less) were discarded from the analysis.

We benchmarked the accuracy of Cappable-seq using the previously mapped TSSs of *fruA* and *mrpC*. For *fruA*, we identified 12 potential TSSs in both biological replicates (see Table S2A in the supplemental material). The potential TSSs at −235 and −286 relative to the first nucleotide in the start codon (from here on, translation start codon [TSC]) were significantly above the threshold and observed at all time points, while the remaining 10 were close to the threshold and generally not observed at all time points. The signal for the TSS at −235 increased during development, while the one at −286 did not (Fig. S2A; Table S2A). A TSS at −235 matched the RNA-seq data (see Fig. S2A in the supplemental material). Importantly, the TSS at −235 matches the previously identified TSS using primer extension on RNA isolated from developing cells (53). For *mrpC*, two potential TSSs were identified (Table S2A). The TSS at −58 bp relative to TSC had the highest score, was detected at all time points in both replicates, and increased during development (Fig. S2B; Table S2A). The potential TSS at −21 relative to the TSC was close to the threshold and detected only at 12 and 24 h. A TSS at −58 matched the RNA-seq data (Fig. S2B). Importantly, a TSS located at −60 bp relative to TSC was identified previously using primer extension on RNA from developing cells (64). We conclude that Cappable-seq reproduces previously identified TSSs of *fruA* and *mrpC* with good accuracy and also identified alternative potential TSSs. These alternative TTSs are likely explained by the higher sensitivity of Cappable-seq than that of primer extension (63). Further work is needed to verify whether they represent genuine TSSs.

10.1128/mbio.00044-22.2 FIG S2 Benchmarking of transcriptional start site mapping using Cappable-seq. (A and B) Analysis of *fruA* and *mrpC* promoter regions with Cappable-seq and RNA-seq. RNA-seq (bottom) and Cappable-seq (top) data are visualized at different time points. For each time point, data for both biological replicates are shown in blue and orange. For Cappable-seq, the RRS is indicated for each TSS on a log_2_ scale; for RNA-seq, RPKM values were calculated for each nucleotide position. The data from RNA-seq and Cappable-seq were obtained from different samples. +1 indicates the TSC. TSSs are indicated in purple relative to the TSC. The center of the MrpC ChIP-seq peak is indicated in brown. Only the highest scoring TSSs are included. For all potential TSSs, see Table S2A. Download FIG S2, EPS file, 1.9 MB. Copyright © 2022 Kuzmich et al. 2022 Kuzmich et al. https://creativecommons.org/licenses/by/4.0/ This content is distributed under the terms of the Creative Commons Attribution 4.0 International license.

10.1128/mbio.00044-22.8 TABLE S2 Combined analysis of transcriptional start sites (TSSs) and MrpC ChIPseq data for *fruA* and *mrpC* (A), genes encoding c-di-GMP-associated proteins (B), and their operons (C).
TSS strand, location of TSS on + or – strand. Max counts 1 and 2, relative read score (RRS) for replicate 1 and 2 − the number of trimmed reads mapping to each position on the genome normalized to the total number of mapped reads was calculated using the following formula: RRS = (Rns/Rt) × 1,000,000 with Rns being the number of trimmed reads mapping to position n in the genome on strand s, and Rt being the total number of reads mapping to the genome. Max counts 1 and 2 indicate the RRS for the max position. Max position, cenome coordinate of the highest TSS position in TSS cluster. Position of TSS relative to gene start, distance (in bp) between max position and gene start of the gene listed in old MXAN and nNew MXAN. MrpC peak coordinate, coordinate of peak summit identified in Chip-Seq analysis (50). Position of MrpC peak relative to gene start, for each peak the distance to the closest gene start codon was calculated and the gene name in new annotation was assigned. Distance between MrpC peak and TSS, distance (in bp) between MrpC peak coordinate and max position of the gene listed in old MXAN and new MXAN. Distance between MrpC peak and TSS ≤ 200 bp, “yes” indicates the gene listed in old MXAN and new MXAN, which MrpC peak coordinate located from max position at the distance of 200 bp or less. Download Table S2, XLSX file, 0.1 MB. Copyright © 2022 Kuzmich et al. 2022 Kuzmich et al. https://creativecommons.org/licenses/by/4.0/ This content is distributed under the terms of the Creative Commons Attribution 4.0 International license.

### MrpC regulates expression of several genes for c-di-GMP-associated proteins during development.

Having validated the Cappable-seq approach, we aimed to identify the transcriptional units encoding c-di-GMP-associated proteins. For this step, we defined genes likely to be in an operon as those transcribed from the same strand and with an intergenic distance between the stop and start codon of flanking genes of ≤50 bp. By combining these data with Cappable-seq data, most genes encoding c-di-GMP-associated proteins could be divided in the following four categories: genes likely not part of an operon (32), likely first gene in an operon (11), likely internal gene in operon (4), and likely internal gene in operon and with an internal promoter (12). For four predicted operons and seven genes predicted not to be in an operon, no TSSs were detected (Table S2B and C).

**FIG 2:**
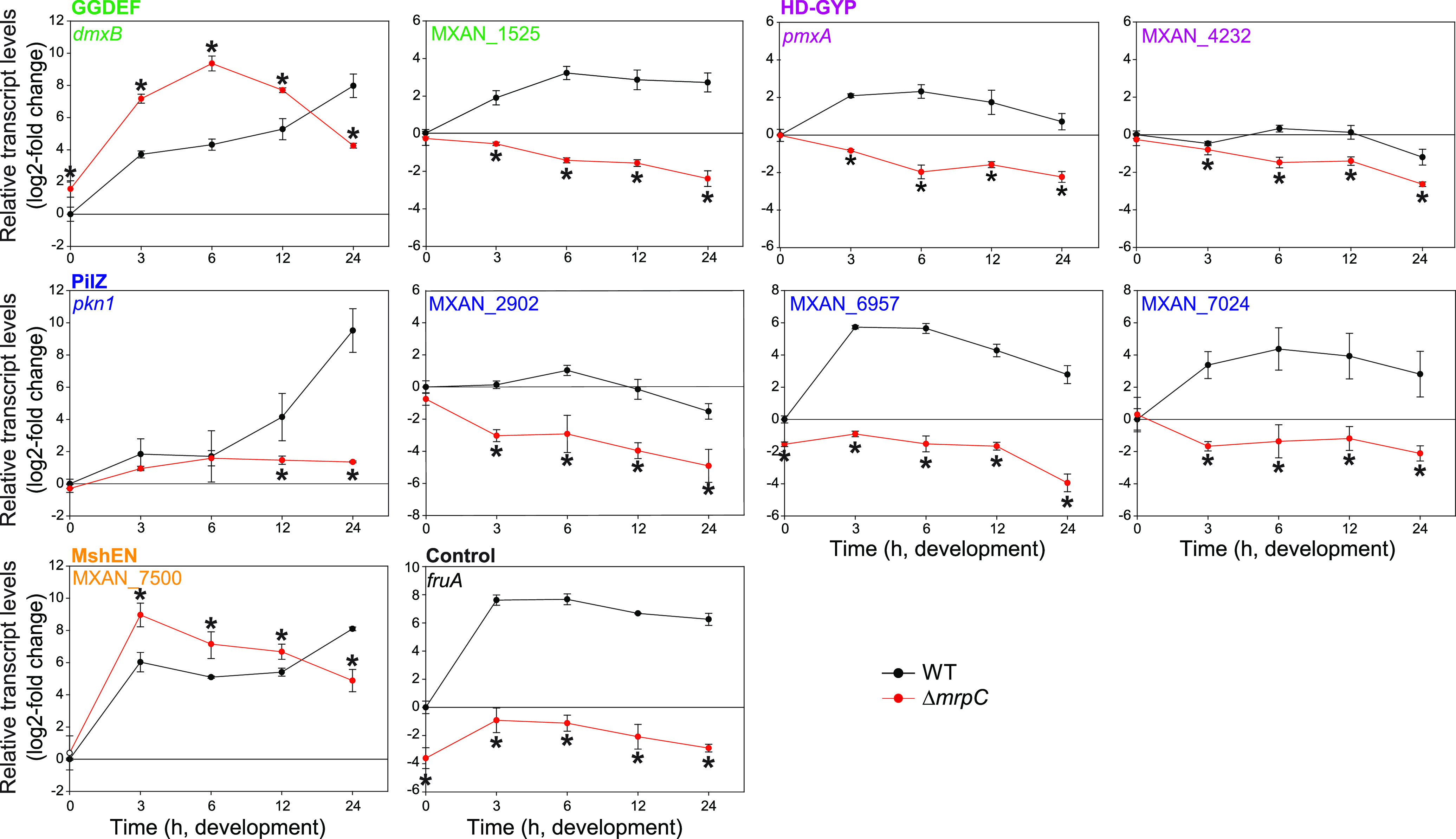
Regulation of the expression of genes encoding c-di-GMP-associated proteins by MrpC. Total RNA was isolated from cells developed in MC7 submerged cultures at the indicated time points from WT (black) and the Δ*mrpC* mutant (red). Transcript levels were determined using RT-qPCR and are shown as mean ± standard deviation (SD) from two biological replicates, each with two technical replicates, relative to WT at 0 h. *, *P* < 0.05; Student’s *t* test in which samples from the Δ*mrpC* mutant were compared with the samples from WT at the same time point. *fruA* served as a positive control. Based on protein sequence analysis, MXAN_1525 and MXAN_4232 are predicted to have DGC and PDE activity, respectively; however, neither a ΔMXAN_1525 nor a ΔMXAN_4232 mutant has defects during growth or development (37, 40). *pkn1*, MXAN_2902, MXAN_6957, and MXAN_7024 are PilZ domain proteins; however, none contain the conserved motifs for c-di-GMP binding (27, 36). Except for Pkn1, a lack of any of these four proteins does not cause defects during growth or development (36, 60). MXAN_7500 is a MshEN domain protein with the sequence motifs for c-di-GMP binding (17); however, it is not known whether this protein binds c-di-GMP or whether it is important during growth and development.

During these analyses, we noticed that several TSSs associated with genes/operons for c-di-GMP-associated proteins were close to binding site(s) for MrpC as mapped at a genome-wide scale using ChIP-seq on cells developed for 18 h (50). That analysis identified >1,500 MrpC binding sites on the M. xanthus genome, of which many map to the promoter regions of developmentally regulated genes. To identify genes/operons for c-di-GMP-associated proteins that could potentially be directly regulated by MrpC, we used two criteria. First, we used the criterion of Robinson et al. (50) who identified promoter regions with an MrpC binding site as those in which the MrpC ChIP-seq peak was located at a distance of −400 to +100 bp from a TSC. Second, based on published experimental data on MrpC binding to the *fruA* and *mrpC* promoters (44, 45, 65), we included the criterion that an MrpC ChIP-seq peak should be located within a distance of 200 bp from a TSS (Fig. S2A and B). Based on these criteria, we identified 18 operons/genes for c-di-GMP-associated proteins that could potentially be regulated by MrpC (Table S2B and C). Using RT-qPCR, we found that 2 (*dmxB* and MXAN_7500) and 7 (MXAN_1525, *pmxA*, MXAN_4232, *pkn1*, MXAN_2902, MXAN_6957, and MXAN_7024) of these 18 genes were expressed at higher and lower levels, respectively, in the Δ*mrpC* mutant than those in the wild type (WT), while 9 genes displayed similar expression patterns in the 2 strains (Fig. 2; see Fig. S3 in the supplemental material). In control experiments, we observed that *gacA* and *gacB*, which are predicted not to be regulated by MrpC (Table S2B), had similar expression levels in WT and the Δ*mrpC* mutant (Fig. S3). The observation that nine of the candidate genes were not expressed in an MrpC-dependent manner under the conditions tested is in agreement with the possibility that the relevant MrpC ChIP-seq peaks may represent false positives as discussed by Robinson et al. (50). We note that the expression of all tested genes in the WT as measured by RT-qPCR matches the expression patterns obtained using RNA-seq (Fig. 1B). The nine differentially expressed genes include six of the most highly developmentally upregulated genes for c-di-GMP-associated proteins (Fig. 1B). These results support that MrpC is a negative regulator of *dmxB* and MXAN_7500 expression and a positive regulator of MXAN_1525, *pmxA*, MXAN_4232, *pkn1*, MXAN_2902, MXAN_6957, and MXAN_7024 expression. From here on, we focused on MrpC regulation of *dmxB* and *pmxA*, which encode enzymatically active proteins that are specifically important for development.

### MrpC negatively regulates *dmxB* expression and DmxB accumulation.

**FIG 3:**
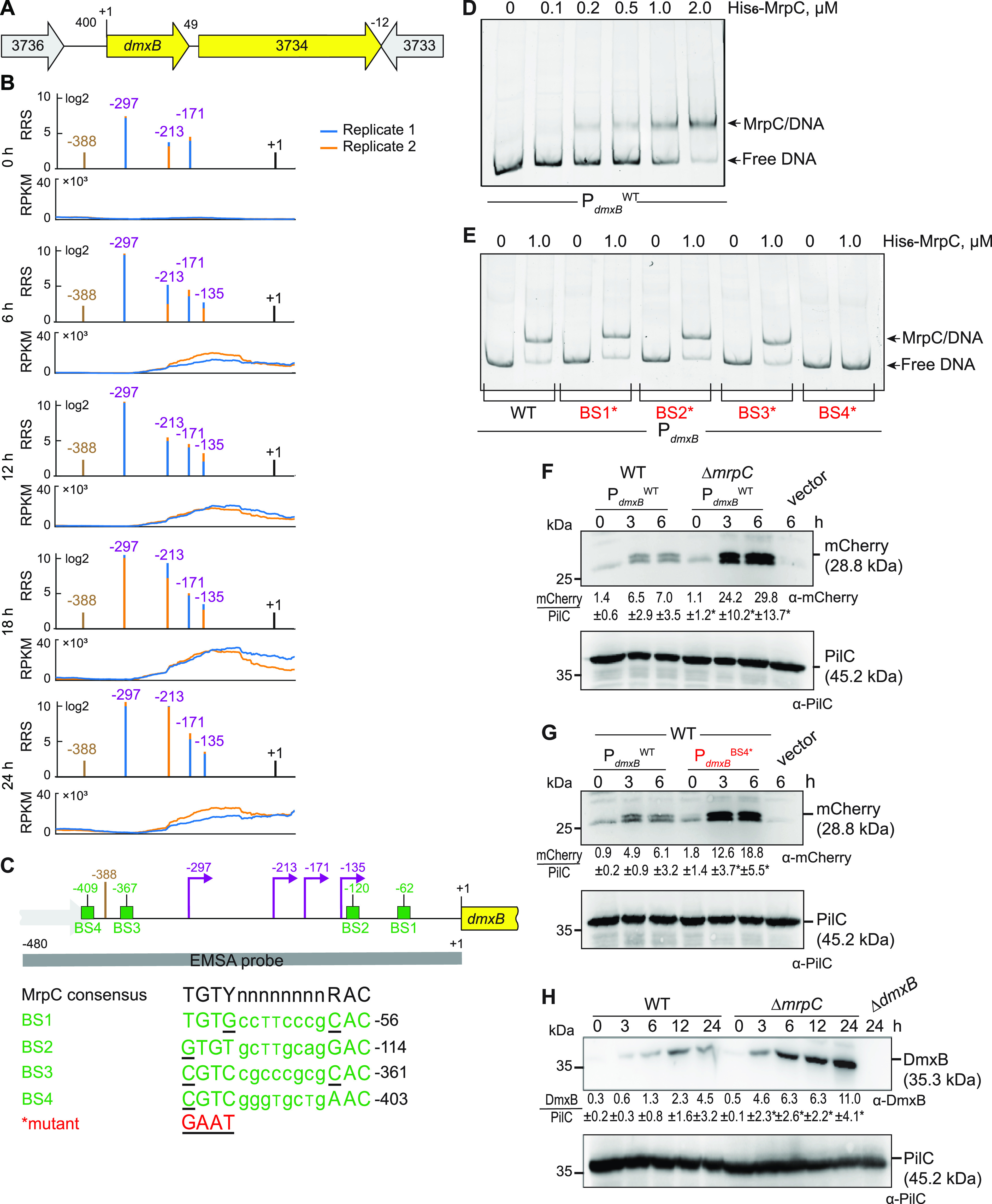
MrpC negatively regulates the expression of *dmxB*. (A) Schematic of the *dmxB* locus. The direction of transcription is indicated by the arrows. +1 indicates TSC of *dmxB*. Numbers above indicate the distance between start and stop codons of flanking genes. MXAN_3734 encodes a 577-amino acid residues protein with a C-terminal receiver domain of response regulators; the remainder of the protein does not contain known domains; MXAN_3734 is not important for development (40). (B) RNA-seq (bottom) and Cappable-seq (top) data at different time points. For each time point, data for both biological replicates are shown in blue and orange. For Cappable-seq, the RRS is indicated for each TSS on a log_2_ scale; for RNA-seq, reads per kilo base per million mapped reads (RPKM) values were calculated for each nucleotide position. Data from RNA-seq and Cappable-seq are from different samples. +1 indicates the *dmxB* TSC. TSSs as mapped by Cappable-seq are indicated in purple relative to the TSC of *dmxB*. The center of the MrpC ChIP-seq peak is indicated in brown. (C) Feature map of *dmxB* promoter region. +1 and color code is as in panel B. Green boxes labeled BS1 to 4 indicate potential MrpC binding sites based on the consensus sequence as defined by reference 50; sequences of BS1 to 4 are shown below and in which underlined text indicates a mismatch. Red indicates the sequence used to generate the mutant binding sites. The gray line indicates the EMSA probe and contains all four predicted MrpC binding sites. (D and E) MrpC binds to the *dmxB* promoter region using BS4. The indicated Hex-labeled probes were mixed with the indicated concentrations of His_6_-MrpC EMSA and analyzed by EMSA. (F) MrpC represses *dmxB* promoter(s). Total cell lysates from the indicated strains expressing *mcherry* from P*_dmxB_*^WT^ were harvested from cells developed in MC7 submerged cultures at the indicated time points. A total of 10 μg of protein was loaded per lane, and samples were separated by SDS-PAGE. Top and bottom blots were probed with α-mCherry and α-PilC antibodies, respectively. The PilC blot served as a loading control. Numbers below the top panel indicate in the accumulation of mCherry relative to PilC as mean ± SD as measured in three biological replicates (Materials and Methods). *, *P* < 0.05; in Student’s *t* test in which samples from the Δ*mrpC* mutant were compared with samples from WT at the same time point. Vector with *mCherry* but without the *dmxB* promoter served as a negative control (vector). mCherry separates into two bands; the reason for this result is not known. (G) BS4 is important for MrpC-dependent repression of *dmxB* promoter(s). Total cell lysates from the indicated WT strains expressing mCherry from the two indicated promoters were prepared and analyzed as in panel F. (H) DmxB accumulates at increased levels in the Δ*mrpC* mutant. Total cell lysates of the indicated strains were harvested from cells developed in MC7 submerged conditions at indicated time points and analyzed as in panel F except that the accumulation of DmxB relative to PilC was calculated.

Based on our criteria as well as RNA-seq, *dmxB* forms a two-gene operon with the downstream gene MXAN_3734 (Fig. 3A; see Fig. S4 in the supplemental material; Table S2B and C). We identified seven potential TSSs upstream of *dmxB* in both replicates (Table S2B and C). Among these TSSs, we focused on four with high scores in both replicates at several time points (Fig. 3B), while the remaining three had low scores and each appeared at only one time point (Table S2B and C). The TSS at −297 relative to TSC was detected with the highest score at all time points and increased as development progressed (Fig. 3B; Table S2B and C). The TSS at −213 was the second highest scoring TSS and sharply increased at 18 h. The TSS at −135 increased slightly during development, while the TSS at −171 did not significantly change in score over time. A comparison of Cappable-seq and RNA-seq data supports that TSSs at −297 and −213 are genuine TSSs (Fig. 3B; Fig. S4). These data support that *dmxB* is transcribed from multiple promoters, and those with TSSs at −297, −213, and −135 are regulated developmentally.

10.1128/mbio.00044-22.4 FIG S4 Mapping of the *dmxB* locus with RNA-seq and Cappable-seq. Top part, *dmxB* locus with numbers above indicating distance between start and stop codons. Bottom part, RNA-seq (bottom) and Cappable-seq (top) data at different time points. For each time point, data for both biological replicates are shown in blue and orange. For Cappable-seq, the RRS is indicated for each TSS on a log_2_ scale; for RNA-seq, RPKM values were calculated for each nucleotide position. The data from RNA-seq and Cappable-seq were obtained from different samples. +1 indicates TSC of gene as shown in the top part. Potential TSSs are indicated in purple relative to the relevant TSC. The center of the MrpC ChIP-seq peak is indicated in brown. Download FIG S4, EPS file, 2.9 MB. Copyright © 2022 Kuzmich et al. 2022 Kuzmich et al. https://creativecommons.org/licenses/by/4.0/ This content is distributed under the terms of the Creative Commons Attribution 4.0 International license.

The *dmxB* promoter region contains an MrpC ChIP-seq peak centered at −388 bp relative to TSC (Fig. 3B and C; Table S2B and C) (50). To test whether MrpC binds directly to the upstream region of *dmxB*, we performed an electrophoretic mobility shift assay (EMSA) using a PCR-amplified 480-bp Hexachloro-fluorescein (Hex)-labeled PCR product that extends from 92 bp upstream of the ChIP-seq peak coordinate to the *dmxB* TSC (Fig. 3C). Titrating purified His_6_-MrpC against the Hex-labeled probe resulted in the formation of one well-defined shifted band consistent with one binding site for MrpC in the *dmxB* promoter region (Fig. 3D).

We identified four potential MrpC binding sites (BS1 to 4) in the *dmxB* promoter region using the consensus sequence defined by reference 50 (Fig. 3C). We prepared four Hex-labeled *dmxB* promoter fragments each containing substitutions of conserved bp in one of the four potential MrpC binding sites as described (44). In EMSAs, the fragments with substitutions in BS1, BS2, or BS3 bound MrpC as the WT fragment (Fig. 3E). In contrast, the fragment with a mutated BS4 did not bind MrpC (Fig. 3E). Based on these data, we suggest that the *dmxB* promoter contains one binding site, i.e., BS4, for MrpC centered at −409 and thus close to the MrpC ChIP-seq peak centered at −388 bp (Fig. 3C).

To test the impact of MrpC and its binding to BS4 on *dmxB* promoter activity *in vivo*, we constructed fluorescent reporters in which the WT *dmxB* promoter fragment (P*_dmxB_*^WT^) used in the EMSAs or the same fragment with a mutated BS4 (P*_dmxB_*^BS4^*) were fused to the start codon of *mCherry* and ectopically expressed from the Mx8 *attB* site. The vector without the *dmxB* promoter served as a negative control. In agreement with the RT-qPCR data (Fig. 2), mCherry expressed from P*_dmxB_*^WT^ accumulated at significantly higher levels in the Δ*mrpC* mutant than that in the WT at all tested time points (Fig. 3F). Importantly, the activity of P*_dmxB_*^BS4^* was significantly higher than that of P*_dmxB_*^WT^ in the WT (Fig. 3G). We conclude that MrpC binds to BS4 to repress *dmxB* expression.

Finally, we observed that DmxB was detected at low levels at 0 h in WT and its accumulation increased during development (Fig. 3H) as previously observed (40). Importantly, DmxB accumulated at significantly higher levels in the Δ*mrpC* mutant than that in the WT during development (Fig. 3H), which is consistent with MrpC acting as a repressor of *dmxB* transcription.

### MrpC positively regulates *pmxA* expression and PmxA accumulation.

**FIG 4:**
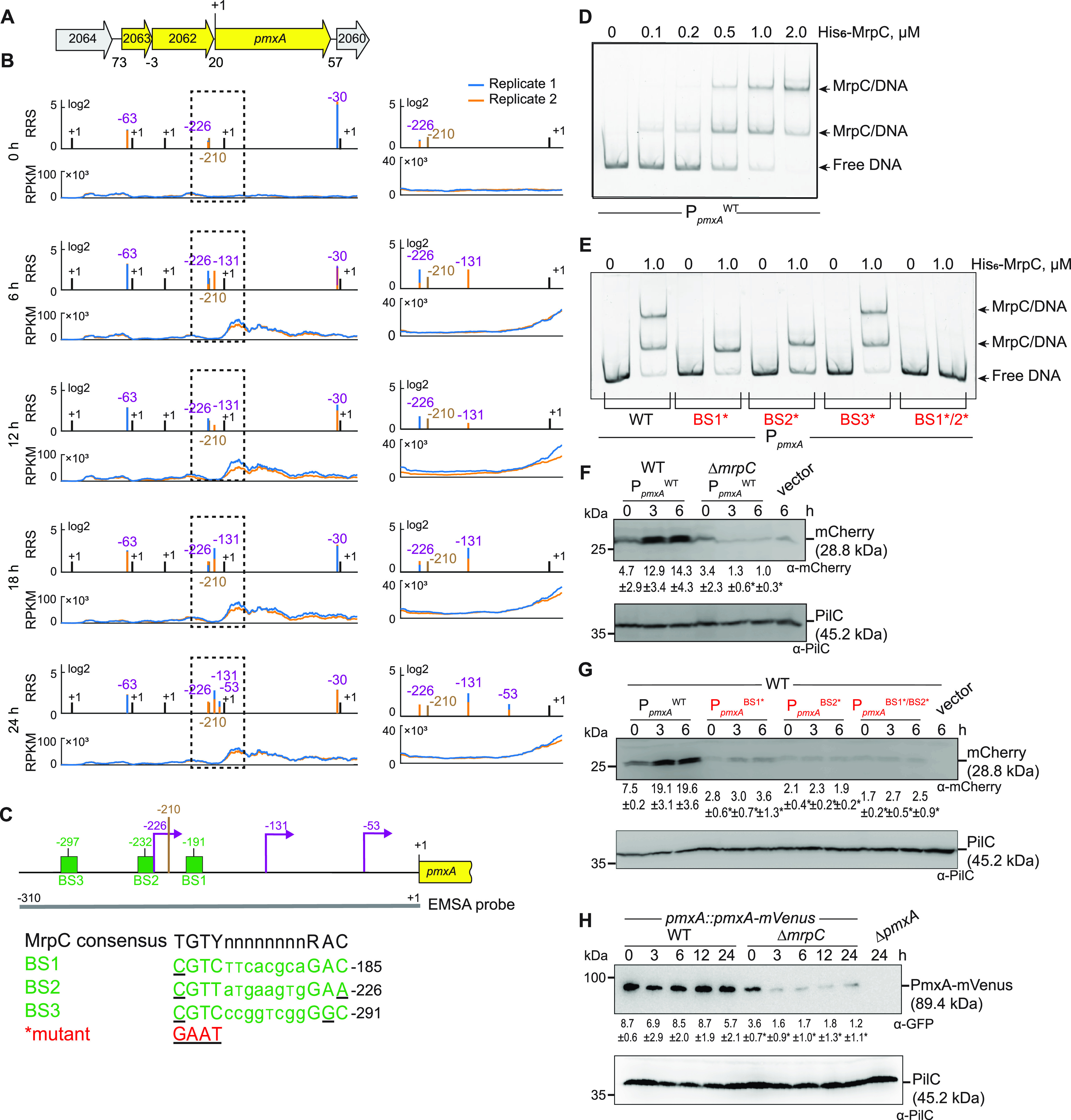
MrpC positively regulates the expression of *pmxA*. (A) Schematic of the *pmxA* locus. The direction of transcription is indicated by the arrows. +1 indicates TSC of *pmxA*. Numbers above indicate the distance between start and stop codons of flanking genes. MXAN_2063 encodes a FecR domain-containing protein with a lipoprotein signal peptide and MXAN_2062 encodes a protein with a type I signal peptide, an N-terminal LysM domain, and a C-terminal extracellular fibronectin type III domain. The function of these two proteins is not known. (B) RNA-seq (bottom) and Cappable-seq (top) data at different time points for genes at *pmxA* locus. For each time point, data for both biological replicates are shown in blue and orange. For Cappable-seq, the RRS is indicated for each TSS on a log_2_ scale; for RNA-seq, RPKM values were calculated for each nucleotide position. The data from RNA-seq and Cappable-seq were obtained from different samples. Left, +1 indicates TSCs of MXAN_2064-_2060; right, zoom of region indicated in the hatched box in left panels immediately upstream of *pmxA* and where +1 indicates the TSC of *pmxA*. In both sets of panels, TSSs as mapped by Cappable-seq are indicated in purple relative to the nearest TSC. The center of the MrpC ChIP-seq peak is indicated in brown. (C) Feature map of *pmxA* promoter region. +1 and color code is as in panel B. Green boxes labeled BS1 to 3 indicate potential MrpC binding sites based on the consensus sequence as defined by reference 50; sequences of BS1 to 3 are shown below and in which underlined text indicates a mismatch. Red indicates the sequence used to generate the mutant binding sites. The gray line indicates the EMSA probe and contains all three predicted MrpC binding sites. (D and E) MrpC binds to the *pmxA* promoter region using BS1 and BS2. The indicated Hex-labeled probes were mixed with the indicated concentrations of His_6_-MrpC EMSA and analyzed by EMSA. (F) MrpC activates *pmxA* promoter(s). Total cell lysates from the indicated strains expressing *mCherry* from P*_pmxA_*^WT^ were harvested from cells developed in MC7 submerged cultures at the indicated time points and then analyzed as in Fig. 3F. (G) BS1 and BS2 are important for MrpC-dependent activation of the *pmxA* promoter(s). Total cell lysates from the indicated WT strains expressing mCherry from the indicated promoters were prepared and analyzed as in Fig. 3F. (H) PmxA accumulates at reduced levels in the Δ*mrpC* mutant. Total cell lysates of the indicated strains were harvested from cells developed in MC7 submerged conditions at indicated time points and analyzed as in Fig. 3F except that the accumulation of PmxA-mVenus relative to PilC was calculated.

Based on our criteria, *pmxA* is the last gene of a three-gene operon (Fig. 4A). Based on Cappable-seq, there is one TSS at −63 relative to the TSC of MXAN_2063 and three TSSs immediately upstream of *pmxA* (Fig. 4B; Table S2B and C). An RT-PCR analysis on RNA isolated from WT at 0 and 6 h of development supports that MXAN_2063-MXAN_2062-*pmxA* is transcribed as an operon at both time points (see Fig. S5A in the supplemental material). The three genes were expressed at a low level at 0 h; at later time points, MXAN_2063 and MXAN_2062 expression remained low, while *pmxA* expression increased (Fig. 4B; Fig. S5B). Accordingly, the score for the single TSS upstream of MXAN_2063 remained low (Fig. 4B; Table S2C). The TSSs upstream of *pmxA* had scores close to the threshold (Table S2B and C). Therefore, we analyzed each biological replicate separately (Fig. 4B, right; Table S2B and C). A TSS at −226 relative to the TSC of *pmxA* was detected at all time points and was not developmentally regulated, while a TSS at −131 was detected at 6 h and later suggested developmental upregulation. A TSS at −53 was detected only at 24 h. We conclude that the MXAN_2063-MXAN-2062-*pmxA* operon is transcribed from a promoter upstream of MXAN_2063 during growth and development; in addition, *pmxA* is transcribed from internal promoters, of which two are developmentally regulated. We identified a single MrpC ChIP-seq peak centered at −210 upstream of the *pmxA* TSC and none upstream of MXAN_2063 (Fig. 4B and C). Consistently, MXAN_2063 and MXAN_2062 expression was independent of MrpC, while *pmxA* expression was significantly decreased in the absence of MrpC during development (Fig. 2; Fig. S5C). Altogether, these observations support that MrpC is specifically involved in the activation of the internal promoter(s) during development.

10.1128/mbio.00044-22.5 FIG S5 Operon mapping of the *pmxA* locus. (A) Schematic of *pmxA* locus and operon mapping. Top, *pmxA* locus in which the direction of transcription is indicated by the arrows. Numbers below indicate distance between start and stop codons of flanking genes; numbers above in purple indicate potential TSSs as mapped by Cappable-seq relative to the nearest TSC. Colored bars below indicate the fragments amplified in the RT-PCR analysis; coordinates indicate the 5′ and 3′ ends of the amplified fragment relative to the relevant start and stop codon. Bottom, agarose gel with results from operon mapping. The fragments indicated in red, orange, green, and blue were amplified from cDNA prepared from total RNA of vegetative (0 h) and developing (6 h) cells, from genomic DNA (gDNA) or from total RNA without addition of reverse transcriptase (RNA). Primer pairs used are as follows: SK318 and SK319 (fragment 2060-*pmxA*), SK320 and SK321 (fragment *pmxA*-2062), SK322 and SK323 (fragment 2062 to 2063), and SK324 and SK325 (fragment 2063 to 2064). (B) Comparison of *pmxA*, MXAN_2063, and MXAN_2062 expression during development in WT. Total RNA was extracted at the indicated points from WT cells developed under MC7 submerged conditions. Transcript levels were quantified using RT-qPCR and are shown as log_2_-fold changes relative to 0 h as mean ± SD from two biological replicates, each with two technical replicates. (C) Comparison of *pmxA*, MXAN_2063, and MXAN_2062 expression during development in WT and the Δ*mrpC* mutant. Total RNA was extracted at the indicated points from WT and Δ*mrpC* cells developed under MC7 submerged conditions. Transcripts levels were quantified using RT-qPCR and are shown as log_2_-fold changes relative to 0 h as mean ± SD from two biological replicates, each with two technical replicates. *, *P* < 0.05; in Student’s t-test in which samples from the Δ*mrpC* mutant were compared with the samples from WT at the same time point. The data for WT are the same as in panel B and the data for *pmxA* are the same as in Fig. 2. (D) Developmental assays for WT, Δ*pmxA* mutant, and WT producing PmxA-mVenus from the native site. Development was performed under MC7 submerged conditions, and cells were imaged at 24 and 120 h. Numbers indicate heat- and sonication-resistant spores at 120 h as a percentage of WT (100%). Scale bar, 100 μm. Download FIG S5, EPS file, 2.2 MB. Copyright © 2022 Kuzmich et al. 2022 Kuzmich et al. https://creativecommons.org/licenses/by/4.0/ This content is distributed under the terms of the Creative Commons Attribution 4.0 International license.

In EMSAs with a 310-bp Hex-labeled probe (Fig. 4C), 0.1 μM His_6_-MrpC gave rise to a single well-defined shifted band, and at 0.5–2.0 μM His_6_-MrpC, an additional well-defined shifted band was evident (Fig. 4D). We identified three potential MrpC binding sites (BS1 to 3) upstream of *pmxA* (Fig. 4C), mutated them separately, and tested His_6_-MrpC binding to the mutated promoters. The P*_pmxA_*^WT^ fragment gave rise to two shifted bands at 1.0 μM His_6_-MrpC, while the fragments containing substitutions in BS1 or BS2 generated only one shifted band, the fragment with substitutions in BS3 behaved as P*_pmxA_*^WT^, and a fragment with both BS1 and BS2 mutated did not bind MrpC at 1.0 μM (Fig. 4E). We conclude that MrpC binds to the internal *pmxA* promoter region at two sites, namely, BS1 and BS2, centered at −191 and −232 relative to the TSC of *pmxA* (Fig. 4C).

The importance of MrpC and its binding to BS1 and BS2 on *pmxA* promoter activity *in vivo* was tested as described for P*_dmxB_* using the same fragments as in the EMSAs. *mCherry* expressed from P*_pmxA_*^WT^ was detected in immunoblots of WT at 0, 3, and 6 h and at significantly reduced levels in the Δ*mrpC* mutant at 3 and 6 h (Fig. 4F), which is in agreement with the RT-qPCR experiments (Fig. 2). Importantly, the activity of P*_pmxA_*^BS1^*, P*_pmxA_*^BS2^*, and P*_pmxA_*^BS1^*^/BS2^* was significantly lower than that of P*_pmxA_*^WT^ in the WT (Fig. 4G).

To determine PmxA levels during development, we used an active PmxA-mVenus fusion (Fig. S5D) expressed from the native site. Surprisingly, the level of PmxA-mVenus did not increase significantly during development in WT (Fig. 4H) despite transcription being upregulated ∼3- to 4-fold during development (Fig. 1B, 2, and 4F). Importantly, the level of PmxA-mVenus in the Δ*mrpC* mutant was reduced significantly compared with that of the WT at all time points (Fig. 4H). Altogether, these observations support that *pmxA* is transcribed from a promoter upstream of MXAN_2063 as well as from internal promoter(s), which are activated by MrpC by binding to BS1 and BS2.

### MrpC curbs accumulation of c-di-GMP and 3′,3′-cGAMP during development.

**FIG 5:**
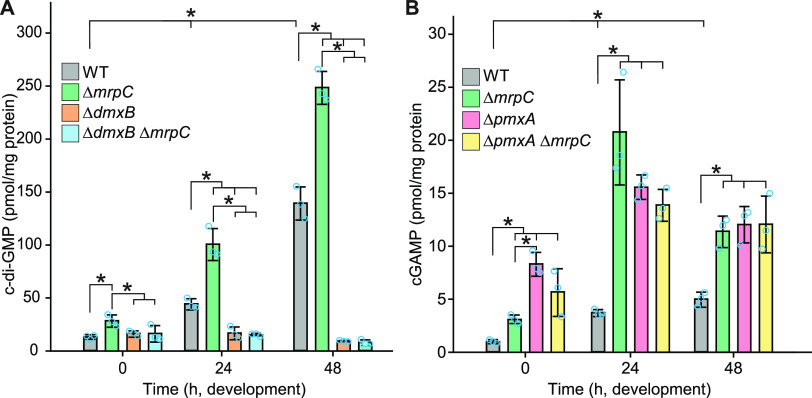
c-di-GMP and cGAMP accumulation during growth and development. (A and B) Cells were harvested at the indicated time points of development, and nucleotide levels and protein concentrations were determined. Levels are shown as mean ± SD calculated from three biological replicates. Individual data points are in light blue. *, *P* < 0.05; in Student’s *t* test. At each specific time point, only pairwise comparisons with significant differences are indicated.

Next, we investigated the functional consequences of the altered accumulation of DmxB and PmxA with respect to cyclic dinucleotides in the Δ*mrpC* mutant. As described (40), the c-di-GMP level increased significantly during development in a DmxB-dependent manner in the WT (Fig. 5A). In overall agreement with the accumulation profile of DmxB, the c-di-GMP level was slightly but significantly higher in the Δ*mrpC* mutant than that in the WT at 0 h and significantly higher during development in the Δ*mrpC* mutant, and the extra c-di-GMP was dependent on DmxB (Fig. 5A).

Because recent studies revealed that PmxA activity against c-di-GMP is significantly lower than toward cGAMP (54), we measured c-di-GMP as well as cGAMP levels in WT and the Δ*pmxA* and Δ*mrpC* mutants. As previously shown (40), the c-di-GMP levels in the WT and the Δ*pmxA* mutant were similar (see Fig. S6 in the supplemental material). The cGAMP level increased significantly during development in WT (Fig. 5B). Importantly, in the Δ*pmxA* mutant, the cGAMP level was significantly higher than in the WT during growth (0 h) as well as development, consistent with the accumulation profile of PmxA-mVenus in WT (Fig. 4H) and PmxA having PDE activity against cGAMP *in vivo*. Consistent with the PmxA-mVenus accumulation profile, the cGAMP level was also significantly higher in the Δ*mrpC* mutant than that in the WT at all time points. Moreover, except at 0 h, cGAMP accumulation in the Δ*mrpC* mutant was not significantly different from that in the Δ*pmxA* mutant. Finally, the Δ*pmxA*Δ*mrpC* double mutant accumulated cGAMP similarly to the Δ*pmxA* mutant. Altogether, these observations support that the increased cGAMP level in the Δ*mrpC* mutant compared with the WT is the result of a decreased accumulation of PmxA.

10.1128/mbio.00044-22.6 FIG S6 c-di-GMP accumulation in WT and the *ΔpmxA* mutant during growth and development. Cells were harvested at the indicated time points of development, and nucleotide levels and protein concentrations were determined. Levels are shown as mean ± SD calculated from three biological replicates. Individual data points are in light blue. *, *P* < 0.05; in Student’s t-test. At each specific time point, no significant differences were observed in pairwise comparisons. Download FIG S6, EPS file, 1.3 MB. Copyright © 2022 Kuzmich et al. 2022 Kuzmich et al. https://creativecommons.org/licenses/by/4.0/ This content is distributed under the terms of the Creative Commons Attribution 4.0 International license.

We conclude that MrpC by regulating the expression of *dmxB* and *pmxA* helps control the cellular pools of c-di-GMP and cGAMP.

### Aggregated and nonaggregated cells accumulate MrpC, DmxB, and PmxA-mVenus as well as c-di-GMP or cGAMP at similar levels.

**FIG 6:**
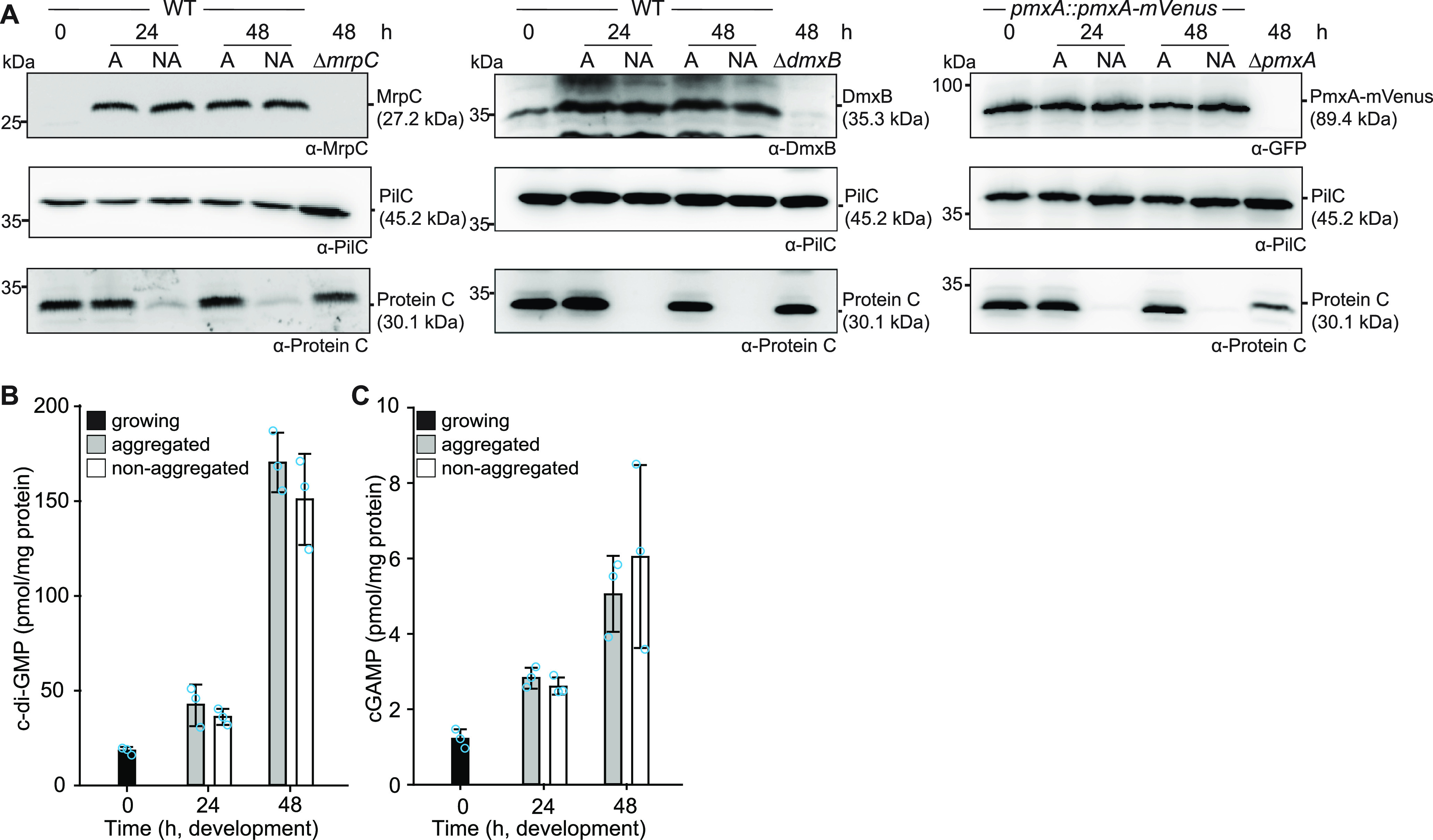
MrpC, DmxA, PmxA-mVenus, c-di-GMP, and cGAMP accumulation in aggregated and nonaggregated cells. (A) MrpC, DmxB, and PmxA-mVenus accumulate at the same levels in aggregated (A) and nonaggregated (NA) cells. Cells were harvested at the indicated time points of development and separated into the two cell fractions. A total of 10 μg of protein was loaded per lane, and samples were separated by SDS-PAGE. Top blots were probed with α-MrpC, α-DmxB, or α-GFP; middle blots with α-PilC; and bottom blots with α-Protein C antibodies. The PilC blots served as loading controls and the Protein C blots as cell separation controls. (B and C) c-di-GMP (B) and cGAMP (C) accumulate at the same levels in aggregated and nonaggregated cells of WT. Samples were generated as in panel A. Levels are shown as mean ± SD calculated from three biological replicates. Individual data points are in light blue. At each specific time point, no significant differences were identified in pairwise comparisons in Student’s *t* test.

In the DZ2 WT strain, MrpC expression and accumulation are higher in aggregated cells, i.e., cells that differentiate to spores within fruiting bodies, than those in nonaggregated cells, i.e., cells that differentiate to peripheral rods (33, 44), raising the possibility that c-di-GMP and/or cGAMP might also accumulate at different levels in these cell types. To this end, we developed DK1622 WT cells under submerged conditions; then separated aggregated and nonaggregated cells at 24 and 48 h of development; and determined MrpC, DmxB, PmxA-mVenus, c-di-GMP, and cGAMP levels in the two cell types. As a control for proper cell separation, we used the accumulation of Protein C, which accumulates in aggregated cells and at a much-reduced level in nonaggregated cells (66). In WT as well as in WT producing PmxA-mVenus, cells were separated properly based on the level of Protein C (Fig. 6A). Surprisingly, at both time points, MrpC accumulated at similar levels in the two cell types (Fig. 6A). Consistently, DmxB and PmxA-mVenus accumulated at similar levels in the two cell types (Fig. 6A), and c-di-GMP (Fig. 6B) as well as cGAMP (Fig. 6C) levels were similar in the two cell types at both time points.

## DISCUSSION

Here, we present a comprehensive analysis of the expression of genes encoding c-di-GMP-associated proteins in M. xanthus. This analysis was motivated by previous observations that the lack of several of these proteins causes defects during only one of the stages of the biphasic life cycle, while others cause defects during both stages. Using RNA-seq, we found that all of these genes were expressed during the life cycle. More importantly, expression of 28 genes encoding c-di-GMP-associated proteins were upregulated, 10 were downregulated, and 32 did not change expression during development. By combining Cappable-seq with data from previously published ChIP-seq analyses of the CRP-like transcription factor MrpC (50), we identified nine genes for c-di-GMP-associated proteins that are regulated (directly or indirectly) by MrpC. Among them, detailed analyses revealed that (i) MrpC binds to and represses the promoter(s) of *dmxB*, which encodes the DGC DmxB that is essential for development and responsible for the dramatic increase in c-di-GMP during development; and (ii) MrpC binds to and activates internal promoter(s) in the MXAN_2063-MXAN_2062_*pmxA* operon to promote the transcription of *pmxA*, which encodes a PDE that is essential for development. Thereby, MrpC regulates the cellular pools of c-di-GMP and cGAMP. Altogether, our findings support that the differential expression of genes for c-di-GMP-associated proteins contributes to their stage-specific function. Moreover, we conclude that MrpC is important for the temporal regulation of genes for c-di-GMP synthesis and cGAMP degradation and a key regulator of cyclic dinucleotide metabolism in M. xanthus.

**FIG 7:**
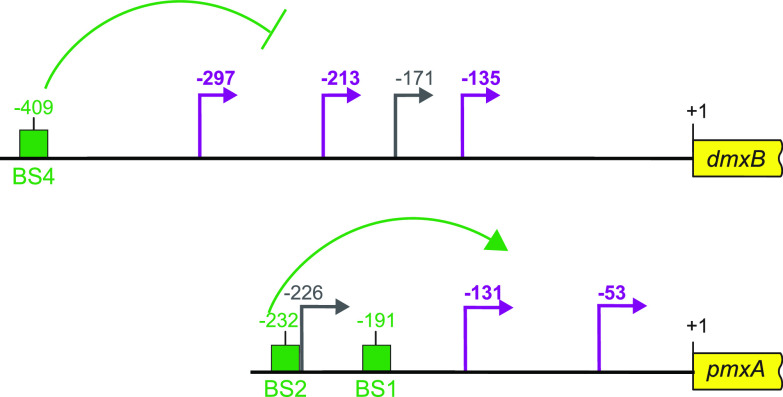
Schematic of *dmxB* and *pmxA* promoter regions. +1 indicate TSC of *dmxB* or *pmxA*; TSSs are indicated in purple and gray and with developmentally regulated TSSs in purple; green boxes indicate verified MrpC binding sites named as in Fig. 3C and 4C. All coordinates are relative to the TSC (+1).

Expression of *dmxB* and DmxB accumulation are upregulated during development (40). Consistently, a lack of DmxB DGC activity causes only developmental defects and no motility defects in the presence of nutrients (37, 40). We found that *dmxB* is likely expressed from four promoters, of which three are developmentally upregulated and one constitutively expressed at a low level (Fig. 7). MrpC is not important for the upregulation of *dmxB* transcription during development; rather, MrpC represses the transcription of *dmxB* during growth and development. Based on EMSAs, MrpC binds to a single site (BS4) centered at −409 relative to the TSC to accomplish this function. The MrpC binding site is located 112, 196, 238, and 274 bp upstream from the four TSSs (Fig. 7); however, from our current analyses, we do not know which promoter(s) is repressed by MrpC. The distance between the MrpC binding sites and the four TSSs strongly argues that MrpC does not directly block the binding of the RNA polymerase. Recently, McLaughlin et al. (44) elegantly demonstrated that MrpC functions as a negative autoregulator of the *mrpC* promoter by outcompeting the binding of the MrpB transcriptional activator, which is an enhancer binding protein. We speculate that MrpC may function by a similar mechanism in *dmxB* expression. However, the activator of *dmxB* developmental expression remains to be identified. The MrpC-dependent repression of *dmxB* expression curbs DmxB synthesis and, consequently, c-di-GMP accumulation slightly during growth and more significantly during development. We previously showed that an increase in the global pool of c-di-GMP is essential for development; however, further increasing this level does not interfere with development (40), arguing that the increased c-di-GMP pool in the Δ*mrpC* mutant may not significantly contribute to the developmental defects in this mutant. Rather, we suggest that the importance of the negative regulation of *dmxB* expression by MrpC lies in avoiding the futile synthesis of DmxB and c-di-GMP.

The lack of PmxA causes only developmental defects and no motility defects in the presence of nutrients (37, 40). Consistently, the expression of *pmxA* is upregulated during development. *pmxA* is the last gene of a three-gene operon, which is expressed a low levels during growth and development. In addition, *pmxA* is expressed from three internal promoters, of which two are developmentally upregulated (Fig. 7). Our data suggest that the developmental upregulation of *pmxA* expression derives from the internal promoters and that MrpC is essential for this upregulation and binds to two sites (BS1 and BS2) centered at −191 and −232 relative to the TSC of *pmxA*. Because BS1 has only one mismatch compared with the consensus MrpC binding site, while BS2 has two (Fig. 4C), we suggest that MrpC binds BS1 with a higher affinity than BS2. From our current analyses, we do not know which of the internal promoters are activated by MrpC. However, based on a comparison to CRP-activated promoters in Escherichia coli (67) and the distance between the MrpC binding sites and the TSSs, we speculate that the promoter with a TSS at −131 relative to the TSC could be activated by MrpC. In the case of the promoter with a TSS at −53, the distance to the MrpC binding sites makes it less likely that this promoter is directly activated by MrpC; however, we notice that CRP in E. coli can act as a structural element from long distances together with an additional transcriptional activator as in the case of the *malK* promoter (68). It is also a possibility that the promoter with a TSS at −53 is activated by MrpC together with FruA as described for several developmentally regulated promoters (46–51). While the transcription of *pmxA* is upregulated during development in the WT, the level of PmxA accumulation (as measured using an active PmxA-mVenus fusion) does not change significantly. In contrast, in the Δ*mrpC* mutant, *pmxA* transcription is not upregulated and PmxA accumulation is strongly decreased. These observations indicate that PmxA accumulation is regulated not only at the transcriptional level but also at the translational and/or posttranslational level. PmxA is essential for development, supporting the idea that the reduced level of PmxA accumulation in the Δ*mrpC* mutant could contribute to the developmental defects in this mutant. However, the developmental defects of the Δ*mrpC* mutant are more severe than those of the Δ*pmxA* mutant (43, 69), supporting that reduced PmxA accumulation alone does not explain the developmental defects in the Δ*mrpC* mutant.

PmxA is a PDE with higher activity toward cGAMP than c-di-GMP (40, 54). Accordingly, the cellular pool of c-di-GMP is unaltered in a Δ*pmxA* mutant compared with that in the WT. We found that the level of cGAMP increased during the development of the WT; importantly, the cGAMP level was significantly higher in the Δ*pmxA* mutant than that in the WT. Similarly, the cGAMP pool was significantly higher in the Δ*mrpC* mutant than that in the WT and similar to that in the Δ*pmxA* mutant during development. These data for the first time show that cGAMP accumulates in M. xanthus *in vivo* and also provide evidence that PmxA is directly involved in its degradation *in vivo*. Neither of the two cGAMP synthases GacA and GacB are important during growth and development (37, 40). Based on RNA-seq and RT-qPCR, *gacA* is upregulated ∼2-fold during development, while *gacB* is constitutively expressed and none of the two genes are regulated by MrpC. We speculate that the increase in cGAMP levels in the WT during development are due to the upregulation of GacA and that the important function of PmxA during development is to maintain this level at an appropriately low level. According to this model, in the Δ*mrpC* mutant, the cGAMP level is increased due to strongly reduced levels of PmxA, while GacA and GacB are likely synthesized as in the WT. In future experiments, it will be interesting to analyze development and the cGAMP level in a Δ*gacA* and Δ*gacB* single and double mutant to determine whether cGAMP is important for development.

In addition to *dmxB* and *pmxA*, MrpC positively or negatively regulates the expression of seven genes for c-di-GMP signaling proteins during development (Fig. 1B, Fig. 2). Among these genes, only the gene for Pkn1, which is upregulated in an MrpC-dependent manner during development, has been shown to be important for development and not for growth (36, 60), suggesting that the lack of Pkn1 may also contribute to the developmental defects in the Δ*mrpC* mutant. Interestingly, we found that some of the MrpC-regulated genes are also differentially expressed during growth. The significance of these observations is not clear because a lack of MrpC was reported to cause only developmental defects (43). Nevertheless, they indicate that MrpC accumulates during growth but has its primary function in development.

The DGC DmxA is important only during growth (37, 40), and its gene is transcriptionally downregulated during development. Based on the mapped MrpC ChIP-seq peaks, this downregulation is independent of MrpC. The reciprocal regulation of *dmxA* and *dmxB* together with the upregulation of *pmxA* support a model whereby the signaling specificity of enzymatically active DGCs and PDE with discrete functions during growth and development rely on their temporally regulated synthesis. In contrast, no clear picture emerges for the experimentally verified c-di-GMP receptors regarding the transcription of the involved genes. The genes for Nla24, SgmT, and PixB that all function during growth and development are constitutively expressed (*nla24* and *sgmT*) or upregulated (*pixB*); the gene for PixA, which functions during growth, is constitutively expressed. Clearly, more work is needed to understand how these receptors are regulated and how their function is restricted to certain stages of the life cycle.

During development, M. xanthus adopts three different cell fates, i.e., peripheral rods, spores, or cell lysis. Previous experiments using the WT strain DZ2 demonstrated that MrpC accumulates in aggregated cells that differentiate to spores but at a much-reduced level in nonaggregated cells that differentiate to peripheral rods (33). Because c-di-GMP drives cell fate determination in Caulobacter crescentus (70), we speculated that c-di-GMP and/or cGAMP could also play a role in cell fate determination in M. xanthus. We found that developing cells of the WT strain DK1622 also segregate into aggregated and nonaggregated cells based on the cell type-specific accumulation of Protein C; however, in this WT strain, MrpC as well as DmxB, PmxA-mVenus, c-di-GMP, and cGAMP accumulated at similar levels in the two cell types. These observations support that a difference in the levels of MrpC, DmxB, PmxA, c-di-GMP, and cGAMP is not involved in determining whether or not cells aggregate during development in DK1622 WT.

## MATERIALS AND METHODS

### Cultivation of M. xanthus and E. coli.

All M. xanthus strains used in this study are derivatives of WT DK1622 (71). In-frame deletions were generated as described (72). All plasmids were verified by sequencing. All strains were confirmed by PCR. M. xanthus strains, plasmids, and oligonucleotides used are listed in Table 1, Table 2, and Table S3 in the supplemental material, respectively. M. xanthus cells were grown at 32°C in 1% CTT (Casitone Tris) broth (1% Bacto Casitone [Gibco], 10 mM Tris-HCl [pH 8.0], 1 mM KPO_4_ [pH 7.6], and 8 mM MgSO_4_) (73) or on 1% CTT 1.5% agar plates with an addition of kanamycin (40 μg · mL^−1^) or oxytetracycline (10 μg · mL^−1^) if relevant. E. coli cells were cultivated in LB (74) or on 1.5% LB agar plates at 37°C with an addition of kanamycin (40 μg · mL^−1^) or tetracycline (10 μg · mL^−1^) if relevant. All plasmids were propagated in E. coli Top10 (Invitrogen Life Technologies) unless otherwise mentioned.

**TABLE 1:** M. xanthus strains used in this study

Strain	Characteristic(s)	Reference
DK1622	Wild type	71
SA5605	Δ*dmxB*	37
SA3546	Δ*pmxA*	37
SA6462	Δ*mrpC*	36
SA8038	*pmxA*::*pmxA*-mVenus	This study
SA8044	Δ*mrpC, pmxA*::*pmxA-mVenus*	This study
SA8096	*attB*::pSK65 (mCherry)	This study
SA8098	*attB*::pSK81 (P*_pmxA_*-mCherry)	This study
SA10108	*attB*::pSK103 (P*_pmxA_*^BS1^*-mCherry)	This study
SA10109	*attB*::pSK105 (P*_pmxA_*^BS2^*-mCherry)	This study
SA10111	*attB*::pSK111 (P*_pmxA_*^BS1^*^/BS2^*-mCherry)	This study
SA8099	*attB*::pSK101 (P*_dmxB_*-mCherry)	This study
SA10110	*attB*::pSK112 (P*_dmxB_*^BS4^*-mCherry)	This study
SA10133	Δ*dmxB* Δ*mrpC*	This study
SA10113	Δ*mrpC attB*::pSK81 (P*_pmxA_*-mCherry)	This study
SA10105	Δ*mrpC attB*::pSK101 (P*_dmxB_*-mCherry)	This study
SA8037	Δ*pmxA* Δ*mrpC*	This study

**TABLE 2:** Plasmids used in this study

Plasmid	Description	Reference
pBJ114	*galK*, Kan^r^	87
pSWU30	*attP*, Tet^r^	88
pPH158	pET28a(+), His_6_-*mrpC*, Kan^r^	33
pSK29	pBJ114, *pmxA*-mVenus, gene replacement at native site, Kan^r^	This study
pSK65	pSWU30, mCherry, *attB*, Tc^r^	This study
pSK81	pSWU30, P*_pmxA_*-mCherry, *attB*, Tc^r^	This study
pSK103	pSWU30, P*_pmxA_*^BS1^*-mCherry, *attB*, Tc^r^	This study
pSK105	pSWU30, P*_pmxA_*^BS2^*-mCherry, *attB*, Tc^r^	This study
pSK114	pSWU30, P*_pmxA_*^BS3^-mCherry, *attB*, Tc^r^	This study
pSK111	pSWU30, P*_pmxA_*^BS1^*^/BS2^*-mCherry, *attB*, Tc^r^	This study
pSK101	pSWU30, P*_dmxB_*-mCherry, *attB*, Tc^r^	This study
pSK121	pSWU30, P*_dmxB_*^BS1^*-mCherry, *attB*, Tc^r^	This study
pSK115	pSWU30, P*_dmxB_*^BS2^*-mCherry, *attB*, Tc^r^	This study
pSK109	pSWU30, P*_dmxB_*^BS3^*-mCherry, *attB*, Tc^r^	This study
pSK112	pSWU30, P*_dmxB_*^BS4^*-mCherry, *attB*, Tc^r^	This study

10.1128/mbio.00044-22.9 TABLE S3 Primers used in this study.
Restriction sites are underlined and mutations introduced by site-directed mutagenesis are in bold. Download Table S3, DOCX file, 0.03 MB. Copyright © 2022 Kuzmich et al. 2022 Kuzmich et al. https://creativecommons.org/licenses/by/4.0/ This content is distributed under the terms of the Creative Commons Attribution 4.0 International license.

### Development under submerged conditions and cell separation.

Exponentially growing M. xanthus in CTT were harvested at 5,000 × *g* for 5 min and resuspended in MC7 buffer (10 mM morpholinepropanesulfonic acid [MOPS; pH 6.8] and 1 mM CaCl_2_) to 7 × 10^9^ cells mL^−1^. A total of 1 mL of concentrated cells was added to 10 mL of MC7 buffer in a polystyrene petri dish with a diameter of 9.2 cm (Sarstedt). For the separation of aggregated and nonaggregated cells during development, cells were developed as described and separated following the procedure of reference 33. Cells were visualized using a Leica DMi8 inverted microscope with a Leica DFC280 camera. To determine sporulation efficiency, cells at 120 h of development were harvested, sonicated for 1 min (30% pulse; 50% amplitude with a UP200St sonifier and microtip; Hielscher) to disperse fruiting bodies, and then incubated at 55°C for 2 h. Sporulation efficiency was calculated as the number of sonication- and heat-resistant spores formed after 120 h of development, relative to the WT. Spores were counted in a counting chamber (depth, 0.02 mm; Hawksley).

### RNA sequencing.

Total RNA from M. xanthus cells developed under submerged conditions was extracted from cells using TRI Reagent (Sigma-Aldrich) according to the manufacturer’s protocol. Purified RNA was treated with a Turbo DNA-free kit (Invitrogen) according to the manufacturer’s protocol. RNA integrity was analyzed by 1% agarose gel electrophoresis. For all samples, rRNA depletion, library preparation, and sequencing were performed at the Max-Planck-Genome-Centre Cologne, Germany. rRNA depletion was conducted with 1 μg total RNA using the Ribo-Zero rRNA removal kit bacteria (Illumina), followed by library preparation with NEBNext ultra directional RNA library prep kit for Illumina (New England BioLabs). Library preparation included 11 cycles of PCR amplification. Quality and quantity were assessed at all steps via capillary electrophoresis (TapeStation; Agilent Technologies) and fluorometry (Qubit; Thermo Fisher Scientific). Sequencing was performed on a HiSeq 3000 instrument (Illumina) with 1 × 150-bp single reads. Libraries were resequenced until a sufficient number of reads were obtained. Sequencing files can be downloaded from EBI ArrayExpress under accession number E-MTAB-11043.

### Cappable-sequencing.

Total RNA was isolated from M. xanthus cells developed under submerged conditions as described. Library preparation and sequencing were performed at Vertis Biotechnologie AG, Freising, Germany as described in reference 63. Briefly, 5′ triphosphorylated RNA was capped with 3′-desthiobiotin-tetraethylene glycol-guanosine 5′ tri-phosphate (DTBGTP) (New England BioLabs) using the vaccinia capping enzyme (VCE) (New England BioLabs). Then, biotinylated RNA molecules were captured using streptavidin beads and eluted with a biotin-containing buffer. RNA samples were poly(A)-tailed using poly(A) polymerase. Then, the 5′-PPP or CAP structures were converted to 5′-P using CAP-Clip acid pyrophosphatase (Cellscript). Afterward, an RNA adapter was ligated to the newly formed 5′-monophosphate structures. First-strand cDNA synthesis was performed using an oligo(dT)-adapter primer and Moloney murine leukemia virus (M-MLV) reverse transcriptase. The resulting cDNAs were PCR amplified using the proof-reading Herculase II fusion DNA polymerase (Agilent). The libraries were amplified in 15 cycles of PCR. The generated cDNA libraries were sequenced on an Illumina NextSeq 500 system using a 75-bp read length. Sequencing files can be downloaded at EBI ArrayExpress under accession number E-MTAB-11042.

### Organism.

The genome and annotation of Myxococcus xanthus DK 1622 (NC_008095.1, downloaded 28 January 2019) were used for all analyses.

### RNA-seq analysis.

All sequencing runs of one sample were concatenated using “cat” (GNU coreutils 8.30). As reverse transcription is part of the sequencing protocol, this was compensated for by “reverse_complement” of the FASTX-Toolkit 0.0.14 (http://hannonlab.cshl.edu/fastx_toolkit). The differential gene expression analysis was done using the RNA-seq pipeline Curare 0.2.1. This software will be described in detail in a separate manuscript. Briefly, the reads were aligned using Bowtie2 2.4.2 in “very-sensitive” mode and with “–mm” option (75). Except for the WT_t24_2 sample, all samples had mapping rates higher than 90% (see Table S4A in the supplemental material). The resulting SAM/BAM files were processed with SAMtools 1.12 (76). The subsequent assignment of mapped reads to genome features was done using the featureCounts (77) of the subread 2.0.1 package (78). featureCounts was run with “-s 1” settings assigning reads in a strand-specific manner to the “gene” features. For every sample, more than 93% of all reads could be assigned to a gene feature (Table S4B). Normalized read counts were calculated and differential gene expression determined with DESeq2 1.30.1 (79). Specifically, DESeq2 automatically calculated the normalized read counts, which were exported via the R (https://www.R-project.org/) commands “dds <- estimateSizeFactors(dds); counts(dds, normalized = TRUE);.” The Curare version of this analysis can be downloaded at Zenodo (doi:10.5281/zenodo.5541852). For coverage plots, BAM files were reads per kilo base per million mapped reads (RPKM) normalized with a bin size of one using the deepTools bamCoverage 3.5.1 (“bs 1 –normalizeUsing RPKM”) (80). The count table and mapping results can be downloaded from EBI ArrayExpress under accession number E-MTAB-11043.

10.1128/mbio.00044-22.10 TABLE S4 Mapping of RNA-seq and Cappable-seq data.
Table S4A. Mapping rates for RNA-seq. Bowtie 2 (Single End, –very-sensitive, –mm, version 2.4.2) mapping rates of RNA-seq samples.
Table S4B. Feature assignment of RNA-seq data. FeatureCounts (Assigned to 'gene' features, Subread version 2.0.1) statistics of M. xanthus DK 1622 RNA-seq data. #Reads: Total number of reads used in FeatureCounts. Assigned: Number of reads assigned to a 'gene' feature. Unassigned (No Feature): Reads, which were aligned to areas without any features. Unassigned (Ambiguity): Reads, which could be assigned to at least two features.
Table S4C. Mapping rates of Cappable-seq samples. Bowtie 2 (Single End, –very-sensitive, –mm, –all, version 2.4.2) mapping rates of Cappable-seq samples. Download Table S4, XLSX file, 0.01 MB. Copyright © 2022 Kuzmich et al. 2022 Kuzmich et al. https://creativecommons.org/licenses/by/4.0/ This content is distributed under the terms of the Creative Commons Attribution 4.0 International license.

### Cappable-seq analysis.

The TSS pipeline in reference 63 was used for TSS detection with modifications. This modified pipeline will be described in detail in a separate manuscript. Briefly, the raw Cappable-seq reads were mapped with Bowtie 2 2.4.1 using “–all,” “–mm,” and “–very-sensitive” settings (75). As in the RNA-seq analysis, all samples except WT_t24_2 had a mapping rate of >90% (Table S4C). A custom script was used to filter all non-best mappings of each read (two equal good mappings will be counted as half a read/mapping each). Created SAM and BAM files were processed using SAMtools 1.12 (76) and Pysam 0.16 (https://pysam.readthedocs.io/). Only the first base of each mapping was used for building “alignments per base” scores (Rns) and every following step. The following formula, altered from reference 63, was used to normalize these scores: RRS = (Rns/Rt) × 1,000,000 (RRS, relative read score; Rt, total number of reads mapped). As in reference 63, an RRS of 1.5 was used as the lower threshold. The first mapped nucleotide from the sequencing reads identifies the orientation and position of the first nucleotide of the primary transcript. TSSs within three nucleotides were clustered into one TSS. In the case of flanking clusters or TSSs within a distance of three or fewer nucleotides, they were merged into one large cluster. The TSS with the highest RRS in a cluster (maximum [max] position) was defined as the major TSS and used in these analyses. The complete pipeline can be downloaded at Zenodo (doi:10.5281/zenodo.5541852). The mapping and TSS results can be downloaded from EBI ArrayExpress under accession number E-MTAB-11042.

### RT-qPCR.

A total of 1 μg of total RNA isolated as described above was used to synthesize cDNA with the high-capacity cDNA reverse transcription kit (Applied Biosystems) according to the manufacturer’s protocol. cDNA templates were diluted 10-fold; 2 μl of a diluted sample was used as a template for RT-qPCR, which contained 1× SYBR green PCR master mix (Applied Biosystems), 2.5 μM of each primer, and H_2_O to a final volume of 25 μL. A 7500 real-time PCR detection system (Applied Biosystems) was used for RT-qPCR measurements using standard conditions. Experiments were done in two biological replicates, each in two technical replicates. Relative gene expression levels were calculated using the comparative threshold cycle (*C_T_*) method.

### Operon mapping.

RNA preparation was done as described. Primers used are listed in Table S3 and used on genomic DNA, RNA without addition of reverse transcriptase, and cDNA.

### Immunoblot analysis.

Immunoblots were carried out as described (74). Rabbit polyclonal α-DmxB (1:1,000 dilution) (40), α-GFP (Roche; 1:2,000 dilution), α-mCherry (Biovision; 1:2,000 dilution), α-protein C (1:2,000 dilution) (81), and α-PilC (1:5,000 dilution) (82) antibodies were used together with horseradish-conjugated goat anti-rabbit immunoglobulin G (Sigma-Aldrich) or anti-mouse sheep IgG antibody (GE Healthcare) as the secondary antibody. Blots were developed using Luminata crescendo Western horseradish peroxidase (HRP) substrate (Millipore) and visualized using a LAS-4000 luminescent image analyzer (Fujifilm). To quantify immunoblots, the signal intensities of individual bands representing the protein of interest and the loading control PilC from the same sample were quantified using Fiji (83); subsequently, the intensity of the band for the protein of interest was normalized relative to the PilC loading control. All immunoblots were performed in three independent biological replicates and mean ± SD calculated.

### Protein purification.

To purify His_6_-MrpC, E. coli Rosseta 2 (DE3)/pLysS strain (Novagen) was transformed with pPH158 (33). The culture was grown in 1 L LB with an addition of chloramphenicol and kanamycin at 37°C to an optical density at 600 nm of 0.5 to 0.7. Protein expression was induced by addition of isopropylthio-β-galactoside (IPTG) to a final concentration of 0.5 mM for 3 h at 37°C. Cells were harvested by centrifugation at 5,000 × *g* for 10 min at 4°C and resuspended in lysis buffer (50 mM NaH_2_PO_4_, 300 mM NaCl, 5 mM MgCl_2,_ 10 mM imidazole, 5% glycerol, [pH 8.0], and complete protease inhibitor cocktail tablet [Roche]). Cells were disrupted using a French press and harvested at 48,000 × *g* for 40 min at 4°C. The cleared supernatant was filtered with a 0.45-μm sterile filter (Millipore Merck, Schwalbach) and applied to a column with 2 mL of Ni^2+^-nitrilotriacetic acid (NTA)-agarose (GE Healthcare) equilibrated with wash buffer (50 mM NaH_2_PO_4_, 300 mM NaCl, 5 mM MgCl_2_, 50 mM imidazole, and 5% glycerol, [pH 8.0]). Protein was eluted with elution buffer (50 mM NaH_2_PO_4_, 300 mM NaCl, 5 mM MgCl_2_, 100 to 500 mM imidazole, and 5% glycerol [pH 8.0]). Fractions containing purified His_6_-MrpC were combined and loaded onto a HiLoad 16/600 Superdex 200-pg (GE Healthcare) size exclusion chromatography column equilibrated with lysis buffer without imidazole. Fractions containing His_6_-tagged MrpC were frozen in liquid nitrogen and stored at −80°C.

### Electrophoretic mobility shift assay (EMSA).

Hex-labeled probes were generated using the primer pairs listed in Table S3 and plasmids containing the WT or mutant promoters as the templates. Assays were performed as described (84). Briefly, purified His_6_-MrpC was mixed at the indicated concentrations with 6 nM (*dmxB* fragments) or 10 nM (*pmxA* fragments) of Hex-labeled DNA fragment in reaction buffer (10 mM Tris [pH 8.0], 50 mM KCl, 1 mM dithiothreitol [DTT], 10 μg · mL^−1^ bovine serum albumin [BSA], 10% glycerol, 0.5 μg herring sperm DNA [Thermo Fisher Scientific]) in a total volume of 10 μl and incubated for 15 min at 20°C. Reaction samples were separated on a 5% polyacrylamide gel in 0.5× Tris-borate-EDTA (TBE; 45 mM Tris, 45 mM borate, and 1 mM EDTA) for 1.5 h. Gels were imaged using a Typhoon phosphoimager (GE Healthcare).

### c-di-GMP and cGAMP quantification.

To quantify the c-di-GMP and cGAMP levels, cells were grown in CTT or developed under submerged conditions as described. Cells were harvested at 2,500 × *g* for 20 min at 4°C and lysed in extraction buffer (high-pressure liquid chromatography [HPLC]-grade acetonitrile-methanol-water [2/2/1, vol/vol/vol]), and supernatants were evaporated to dryness in a vacuum centrifuge. Pellets were dissolved in HPLC-grade water and analyzed by liquid chromatography-tandem mass spectrometry (LC-MS/MS). c-di-GMP and cGAMP quantification were performed at the Research Service Centre Metabolomics at the Hannover Medical School, Germany. Experiments were done in three biological replicates. Protein concentrations were determined in parallel using a Pierce microplate bicinchoninic acid (BCA) protein assay kit (Thermo Scientific).

### Bioinformatics.

Heatmaps were created using R package pheatmap (https://cran.r-project.org/web/packages/pheatmap/index.html). Protein domains were identified using Pfam v33.1 (pfam.xfam.org) (85); signal peptides were predicted with SignalP 5.0 (https://services.healthtech.dtu.dk/service.php?SignalP-5.0) (86).

### Data availability.

All data supporting this study are available within the article, the supplemental information files, or at EBI Arrayexpress (http://www.ebi.ac.uk/arrayexpress; RNA-seq, E-MTAB-11043; Cappable-Seq, E-MTAB-11042). Code for the Cappable-seq analysis and the Curare version used for the RNA-seq analysis can be found at Zenodo (https://www.zenodo.org, ID: 5541852).
