# Supplementary material for: CRP-Like Transcriptional Regulator MrpC Curbs c-di-GMP and 3′,3′-cGAMP Nucleotide Levels during Development in Myxococcus xanthus
Source: mBio. 2022 Feb 15;13(1):e00044-22. doi: 10.1128/mbio.00044-22 (PMC8844925; doi:10.1128/mbio.00044-22)
Supplement: TABLE S3 [file mbio.00044-22-st003.docx]

**Table S3.** Primers used in this study

| Name | Sequence (5’→3’) | Description |
| --- | --- | --- |
| SK44 | GCGCGGTACCAGATGAAGCTCACCGAGC | Endogenous substitution of *pmxA* to *pmxA*-mVenus |
| SK47 | GCGCTCTAGAGGCCGCTCGAATCCCTCCG |  |
| SK83 | CTTCGGCCGTTACTTGTACAGCTCGTC |  |
| SK84 | TACAAGTAACGGCCGAAGGATGGTAGG |  |
| mCherry_R XbaI | ATCGTCTAGATTACTTGTACAGCTCGTCCATGCC | mCherry |
| SK279 | CGGCGAATTCTCCGCCGCCGGTCATGCC | Cloning of P*dmxB* mCherry |
| SK280 | AATGAAGCGGAGTGGTGCATGGTGAGCAAGGGCGAG |  |
| SK281 | CTCGCCCTTGCTCACCATGCACCACTCCGCTTCATT |  |
| SK355 | GTTGTGGGCGTTGGG**TAAG**GCTTGCAGGACCGGG | P*dmxB* BS1 site directed mutagenesis |
| SK356 | CCCGGTCCTGCAAGC**CTT**ACCCAACGCCCACAAC |  |
| SK343 | AGGGGCGTTGGGAGC**CTTA**GGAAGGGCGTGGCAC | P*dmxB* BS2 site directed mutagenesis |
| SK344 | GTGCCACGCCCTTCC**TAAG**GCTCCCAACGCCCCT |  |
| SK304 | CGCGCGGCCACCGAG**GAAT**CGCCCGCGCACGGGAATATCTAC | P*dmxB* BS3 site directed mutagenesis |
| SK305 | GTAGATATTCCCGTGCGCGGGCG**ATTC**CTCGGTGGCCGCGCG |  |
| SK316 | TGCAGTTCAGCACCC**ATTC**CTAGCGACCCGTCGC | P*dmxB* BS4 site directed mutagenesis |
| SK317 | GCGACGGGTCGCTAG**GAAT**GGGTGCTGAACTGCA |  |
| SK276 | CCCTGGAGGAGCCACGCCATGGTGAGCAAGGGCGAG | Cloning of P*pmxA* mCherry |
| SK277 | CTCGCCCTTGCTCACCATGGCGTGGCTCCTCCAGGG |  |
| SK278 | CGGCGAATTCTGAAGCGACGCCCCGACCGG |  |
| SK300 | CAGGAGCAACGCGC**GAAT**TTCACGCAGACAGTC | P*pmxA* BS1 site directed mutagenesis |
| SK301 | GACTGTCTGCGTGAA**ATTC**GCGCGTTGCTCCTG |  |
| SK308 | TACGGGGCGCGGAA**GAAT**ATGAAGTGGAAGTGT | P*pmxA* BS2 site directed mutagenesis |
| SK309 | ACACTTCCACTTCAT**ATTC**TTCCGCGCCCCGTA |  |
| SK345 | CGACGCCCCGACCGG**ATTC**GGCACCTGGGCCCGG | P*pmxA* BS3 site directed mutagenesis |
| SK346 | CCGGGCCCAGGTGCC**GAAT**CCGGTCGGGGCGTCG |  |
| 4361_qPCR_forw | CACCGACAAGCGCAAGCAG | RT-qPCR, *cdbA* |
| 4361_qPCR_rev | GCACGACCCAGGAAAGGGA |  |
| 4362_qPCR_forw | CCGAGGACATGCTGGAGGAG | RT-qPCR, *cdbB* |
| 4362_qPCR_rev | CGTTGACCGACGGCATCTTC |  |
| qdmxAf | GTTTCGAGGACAACAACGGG | RT-qPCR, *dmxA* |
| qdmxAr | AAGATCTTCAGCGCACCCAG |  |
| q3735f | GGTCCCTTCTGCTCATCATC | RT-qPCR, *dmxB* |
| q3735r | AGGAACCTGTCCAGGAGGA |  |
| fruA for | ATCATCTCGCAGTGCTTCGA | RT-qPCR, *fruA* |
| fruA rev | CCTCGGACCAGGGAGTTGA |  |
| qmrpcCf | CCGCACAACTCGACCATCTA | RT-qPCR, *mrpC* |
| qmrpcCr | GGTGCTGTTCTTGCCGATG |  |
| 7440 qPCR forw | GTGGACTTCCTCTGCGAATC | RT-qPCR, *nla24* |
| 7440 qPCR rev | GATGACCAGGTCGAAGGACT |  |
| q1087f | AGCTGCACGGAAAGGTCCCC | RT-qPCR, *pixA* |
| q1087r | AGGGCGAAGCCGTCCATCAC |  |
| q1467f | CCCTGTCGGTCATGTGTGAA | RT-qPCR, *pkn1* |
| q1467r | ACGAACTGGACGCCGAAG |  |
| pmxA qPCR fw | GGTGGACATCAAGCAGAAGA | RT-qPCR, *pmxA* |
| pmxA qPCR rev | CCTTCTCCAGGCTCTCGTAG |  |
| q4445f | ACCGCATCATCCCGCTTTAT | RT-qPCR, *tmoK* |
| q4445r | ACACGCTCATGATGGGGAA |  |
| q0961f | TGGTGATGTGGGCTGCTGGG | RT-qPCR, MXAN_0961 |
| q0961r | CCAAGGCGGAGCGAGATGCT |  |
| q1525f | CACCAACGGTACCTTCCTCAA | RT-qPCR, MXAN_1525 |
| q1525r | GATGGTCTCGTGGTACTGGG |  |
| q2649f | TCACGCCGTTGATTGAGATGA | RT-qPCR, MXAN_2649 |
| q2649r | GCAAGTAGAAGGTGCTCTCCA |  |
| q2902f | TGGACGAAATCGAGAACACCA | RT-qPCR, MXAN_2902 |
| q2902r | TGAGCCGGTAGAAGAGGTCC |  |
| q2997f | TCAAGGACGAGTTGGAGGAC | RT-qPCR, MXAN_2997 |
| q2997r | ATGGAGAACTCGGGGATGC |  |
| q3788f | GGCTTGTCGGTGTTGATGTA | RT-qPCR, MXAN_3788 |
| q3788r | AACACCTCGACCCCTCATCC |  |
| q4232f | TGCACGACATCGGGAAGATT | RT-qPCR, MXAN_4232 |
| q4232r | CGGAATGGCCTGGATCATCT |  |
| q6863f | AGAGCAAGCAGAAGCGCGGA | RT-qPCR, MXAN_6863 |
| q6863r | CCAGCTCCTCGACGCGATCC |  |
| q6957f | GTGTGGTCATCGCATCCTCT | RT-qPCR, MXAN_6957 |
| q6957r | CGCCTTCCCGAGACAACA |  |
| q7024f | TCTTCACGGTGGTGGAGTTC | RT-qPCR, MXAN_7024 |
| q7024r | ACACCTTCAGCGTCAGCC |  |
| q7500f | CAGCTCTACCGGGGTGAAAC | RT-qPCR, MXAN_7500 |
| q7500r | GGAGCCTCCATGTTCGTCAG |  |
| SK318 | CGCTTGTTCGTCCATTCGTC | *pmxA* operon mapping |
| SK319 | GGGAAGATTGGCATCGTGGA |  |
| SK320 | CCGAGGAAGATTTCGGCCTT |  |
| SK342 | GTCCGCGTGGAGGCCGAG |  |
| SK322 | CCGTCTCCAGGGCCTTCTGC |  |
| SK323 | GGGCGGCGTCCAAATCAAGC |  |
| SK324 | TGTCATTCTCGTAGAGCGGC |  |
| SK325 | TCTGCTACGACCTGCGATTC |  |
| ePmxA-HEX_F | TGAAGCGACGCCCCGACC | Amplification of Hex-labeled P*pmxA* EMSA probe |
| SK351_ R | GGCGTGGCTCCTCCAGGGGT |  |
| eDmxB-HEX_F | TCCGCCGCCGGTCATGCC | Amplification of Hex-labeled P*dmxB* EMSA probe |
| SK352_R | GCACCACTCCGCTTCATTCG |  |

* Restriction sites are underlined and mutations introduced by site-directed mutagenesis are in bold.
